# Benzophenone Derivatives with Histamine H_3_ Receptor Affinity and Cholinesterase Inhibitory Potency as Multitarget-Directed Ligands for Possible Therapy of Alzheimer’s Disease

**DOI:** 10.3390/molecules28010238

**Published:** 2022-12-28

**Authors:** Justyna Godyń, Paula Zaręba, Dorota Stary, Maria Kaleta, Kamil J. Kuder, Gniewomir Latacz, Szczepan Mogilski, David Reiner-Link, Annika Frank, Agata Doroz-Płonka, Agnieszka Olejarz-Maciej, Sylwia Sudoł-Tałaj, Tobias Nolte, Jadwiga Handzlik, Holger Stark, Anna Więckowska, Barbara Malawska, Katarzyna Kieć-Kononowicz, Dorota Łażewska, Marek Bajda

**Affiliations:** 1 Department of Physicochemical Drug Analysis, Jagiellonian University Medical College, Medyczna 9 St., 30-688 Krakow, Poland; 2 Doctoral School of Medical and Health Sciences, Jagiellonian University Medical College, św. Łazarza 16 St., 31-530 Krakow, Poland; 3 Department of Technology and Biotechnology of Drugs, Jagiellonian University Medical College, Medyczna 9 St., 30-688 Krakow, Poland; 4 Department of Pharmacodynamics, Jagiellonian University Medical College, Medyczna 9 St., 30-688 Krakow, Poland; 5 Institute of Pharmaceutical and Medicinal Chemistry, Heinrich Heine University Duesseldorf, Universitaetsstr. 1, 40225 Duesseldorf, Germany

**Keywords:** multitarget-directed ligands, benzophenone derivatives, Alzheimer’s disease, cholinesterase inhibitors, histamine H_3_ receptor ligands

## Abstract

The multitarget-directed ligands demonstrating affinity to histamine H_3_ receptor and additional cholinesterase inhibitory potency represent a promising strategy for research into the effective treatment of Alzheimer’s disease. In this study, a novel series of benzophenone derivatives was designed and synthesized. Among these derivatives, we identified compound **6** with a high affinity for H_3_R (*K_i_* = 8 nM) and significant inhibitory activity toward BuChE (IC_50_ = 172 nM and 1.16 µM for *eq*BuChE and *h*BuChE, respectively). Further in vitro studies revealed that compound **6** (4-fluorophenyl) (4-((5-(piperidin-1-yl)pentyl)oxy)phenyl)methanone) displays moderate metabolic stability in mouse liver microsomes, good permeability with a permeability coefficient value (P_e_) of 6.3 × 10^−6^ cm/s, and its safety was confirmed in terms of hepatotoxicity in the HepG2 cell line. Therefore, we investigated the in vivo activity of compound **6** in the Passive Avoidance Test and the Formalin Test. While compound **6** did not show a statistically significant influence on memory and learning, it showed analgesic properties in both acute (ED_50_ = 20.9 mg/kg) and inflammatory (ED_50_ = 17.5 mg/kg) pain.

## 1. Introduction

Memory and other cognitive functions such as learning, perception, attention, or decision-making play a crucial role in the everyday life of humans. They are driven by the complex, synergistic interactions between diverse neurotransmitters, i.e., acetylcholine (ACh), histamine, serotonin, glutamate, noradrenaline and many others. The dysfunctions of central nervous system (CNS) mediators and lack of synergism in their cooperation are proven to be involved in the progression of cognitive decline, often resulting in degenerative disorders such as Alzheimer’s disease (AD) [1]. Therefore, a number of therapeutic possibilities for AD therapy are concentrated on neurotransmission enhancement. In 2021 over 20% of phase III clinically tested agents were focused on neurotransmitters, being defined as symptomatic cognitive enhancers [2].

Acetylcholine and histamine are neurotransmitters that play a significant role in cognition and memory processes [3,4,5]. Blockade of the histamine H_3_ receptors (H_3_R) may lead to the stimulation of the histaminergic system (autoreceptors) or among others, to the improvement of the cholinergic neurotransmission (heteroreceptors) [6,7,8]. Inhibition of cholinesterases (enzymes involved in the hydrolysis of ACh within the synapses) results in the stimulation of the cholinergic system [9]. In turn, the increased ACh level within the brain regions related to memory and learning possibly leads to the improvement of cognitive functions [10,11]. Moreover, a decrease in cholinesterase activity may, to some extent, disrupt the amyloid-beta (Aβ) aggregation, a hallmark peptide of AD [5], also making causative treatment of the disease potentially possible.

Therefore, many therapeutic approaches for AD are based on histamine H_3_R antagonists/inverse agonists along with cholinesterase inhibitors [12,13,14]. The research compounds acting as cholinergic enhancers by inhibition of cholinesterases have been clinically tested since the late 1990s [15,16,17] and are still present in clinical trials [2]. Currently used symptomatic drugs for AD treatment, i.e., donepezil and rivastigmine, act as inhibitors of acetylcholinesterase (AChE) and/or butyrylcholinesterase (BuChE) [18]. Additionally, histamine H_3_R antagonists/inverse agonists have been of great interest to researchers; some have reached preclinical and clinical development stages [10,19].

The complexity of neurotransmitter interactions involved in the physiological background of cognitive functions results in the complex and multifactorial pathophysiologies of diseases related to memory. Thus, the research and possible therapies need to focus on such interactions and take into consideration many diverse dysfunctions and mechanisms involved in cognitive decline. Multitarget therapeutic strategies based on multitarget-directed ligands (MTDLs) seem to fulfill such requirements [20,21]. MTDLs are compounds that affect more than one pathophysiological pathway and can be rationally designed as structures with pharmacophore moieties that engage with receptors, enzymes, or other pharmacologically important targets [22].

Regarding neurotransmission, MTDLs designed as promising future AD therapeutics may combine anticholinesterase activity with antagonism/inverse agonism toward H_3_ histamine receptors (H_3_R) in one molecule, thus affecting cholinergic and histaminergic interneuronal communication [13,23]. Such multitarget compounds have been successfully designed and developed recently [24,25].

Our previous studies were focused on MTDLs affecting neurotransmission, mainly cholinergic and histaminergic ones as possible cognitive enhancers [26,27,28]. In addition, some of the developed compounds were found to be promising monoamine oxidase B (MAO B) inhibitors [29,30].

**Figure 1:**
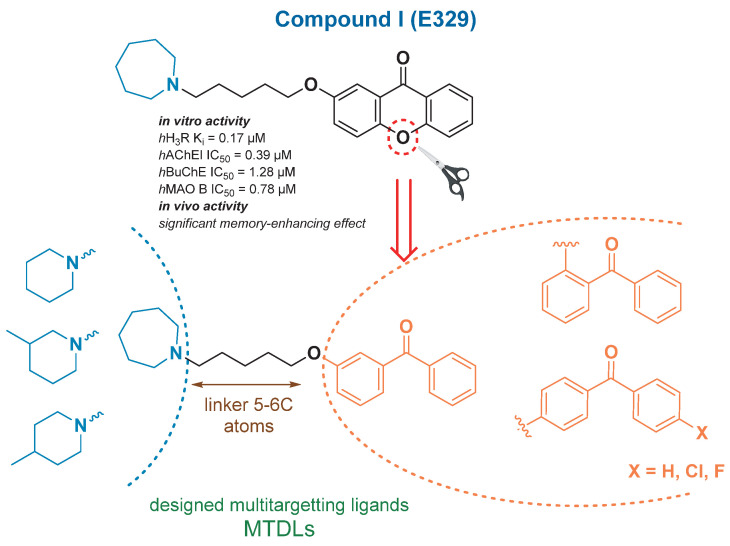
The general structures of the novel compounds designed as H_3_R/ChE/MAO B MTDLs, based on previously obtained [32] promising lead **I**.

The present study was focused on the continuation of the search for MTDLs affecting cholinergic and histaminergic neurotransmission based on our previous findings in the field. In 2018, promising results were published for novel compounds based on a benzophenone scaffold as human H_3_R (*h*H_3_R) antagonists with in vivo anticonvulsant activity [31]. Later, in 2020 structurally related xanthone derivatives were reported combining significant activity toward selected targets, i.e., H_3_R, cholinesterases, and MAO B enzyme with in vivo confirmed memory-enhancing effects [32]. The lead structure **I** developed in that research is presented in Figure 1.

Regarding the obtained results, we decided to continue our research on the potent benzophenone-based H_3_R inhibitors [31], and we designed a series of novel derivatives of benzophenone, structurally related to previously obtained lead **I** (Figure 1). All the compounds intended to interact with H_3_R and cholinesterases along with possibly possessing additional activity toward MAO B. The structures combined a basic tertiary amine and lipophilic aromatic benzophenone moiety connected by an ether linker, structural motifs essential for H_3_R antagonists/inverse agonists [33,34], common also in cholinesterase inhibitors [23,35].

Initially, we evaluated the binding mode of the designed compounds with H_3_R, using H_3_R homology model, and with AChE and BuChE by docking to the enzyme active sites (*electric ray* AChE and *human* BuChE). Then, the biological activity of the synthesized novel MTDLs toward selected targets was assessed in vitro. The designed ligands were screened for their potency as AChE and BuChE inhibitors as well as for affinities to H_3_R. Due to the series origin, the activity toward MAO B was also tested. The most potent compounds were evaluated for their *K_i_* (H_3_R) or IC_50_ values (AChE, BuChE). In further in vitro kinetic studies, selected compounds were investigated to determine the mechanism of AChE and BuChE inhibition. Additionally, regarding the MAO B inhibitory activity of the lead **I** and its derivatives, the binding mode of the obtained compounds within the human MAO B (*h*MAO B) active site has been investigated.

Moreover, for the selected lead compound, the metabolic stability, neuroprotective properties, and potential hepatotoxicity were studied; the permeability of the blood-brain barrier (BBB) in vitro was also determined. Finally, to confirm the therapeutic potential of the lead compound on memory and learning, and its potential analgesic properties, in vivo pharmacological tests were carried out.

## 2. Results and Discussion

### 2.1. Rational Design

Following the general structural requirements for H_3_R, cholinesterases and MAO B affinity, the design of novel MTDLs was rationally performed. Novel derivatives were based on the substituted benzophenone moiety acting as an arbitrary lipophilic region, which was linked by an alkoxyl chain to a (methyl)piperidine or an azepane basic residue. The benzophenone scaffold was a structural analog of the xanthone moiety present in compound **I** [32] (Figure 1).

**Figure 2:**
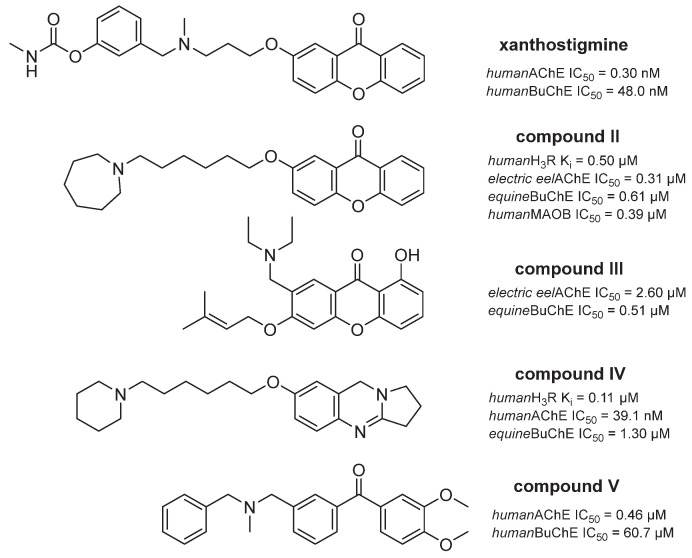
Xanthone, benzophenone and basic tertiary amine motifs present in compounds developed as potential AD therapeutics [13,32,36,37,40].

Xanthone, as well as benzophenone fragments, were found to be pharmacophores for the H_3_R antagonism/inverse agonism [31,32]. Both elements are also important for cholinesterases and/or MAO B inhibition [36,37,38,39,40] (Figure 2). Additionally, basic cyclic tertiary amine moieties with an alkyl linker along with a lipophilic group are known structural characteristics present in MTDLs with inhibitory potency toward AChE and/or BuChE, and with H_3_R affinity [13,40] (Figure 2).

### 2.2. Synthesis of Designed Benzophenones

**Scheme 1:**
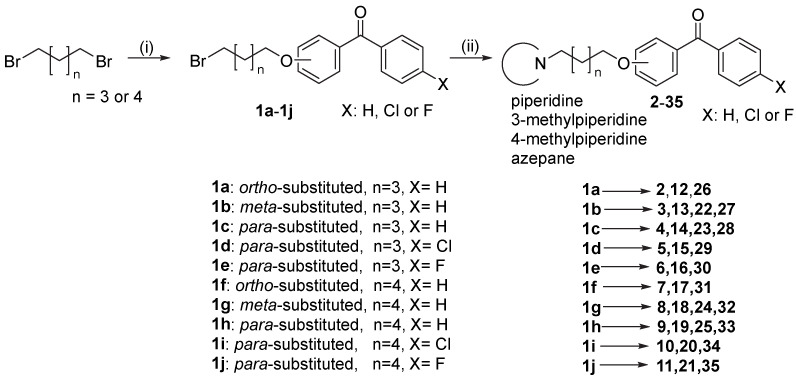
The synthetic pathway of target compounds **2**–**35**. (i) a suitable benzophenone derivative, K_2_CO_3_, KI, acetone, reflux, 10–12 h; (ii) a suitable amine, K_2_CO_3_, KI, ethanol:water (4:1), reflux 12–24 h.

Synthesis of final compounds (**2**–**35**) began with the *O*-alkylation of a suitable benzophenone derivative with dibromopentane or dibromohexane in acetone (Scheme 1). The obtained bromides (**1a**–**1j**) were further used in the *N*-alkylation of corresponding amines (piperidines or azepane) in the mixture of ethanol-water (4:1) and the presence of K_2_CO_3_ [32]. The purity and identity of final compounds were confirmed by spectral (^1^H and ^13^C NMR, LC-MS) and elemental analysis.

### 2.3. In Vitro Activity of Tested Benzophenones

#### 2.3.1. Histamine H_3_ Receptor Affinity

Final compounds were tested for their H_3_R affinity in a radioligand binding assay using [^3^H]*N*^α^-methylhistamine as radioligand in HEK293 cells stably expressing *h*H_3_R [25]. Results are collected in Table 1. All the compounds revealed high affinities for H_3_R, with *K_i_* values ranging from 8 nM (lead **6**) to 371 nM (**2**). The position of the alkoxyl chain in the benzophenone scaffold was important for the effect at the H_3_R. *Para*-substituted derivatives were generally the most potent. Their *ortho* and *meta*-analogs presented weaker H_3_R affinity; however, the latter were usually slightly better than the former, similarly in both subgroups of five and six carbon atoms in the alkoxyl chain (e.g., **4** vs. **3** vs. **2** and **9** vs. **8** vs. **7**; **14** vs. **13** vs. **12** and **19** vs. **18** vs. **17**). The number of methylene groups in the ether linker affected H_3_R affinity. Six carbon atom linker was preferable for *meta* and *ortho*-substituted benzophenones (e.g., **22** vs. **24** and **26** vs. **31**). In turn, five carbon atoms in the alkoxyl part were usually better in the case of *para*-substituted derivatives, especially with additional halogen substituents in the second phenyl ring present in the benzophenone scaffold (e.g., **6** vs. **11**, **16** vs. **21** and **29** vs. **34**). Finally, it seemed that the kind of basic tertiary amine moieties (in the case of target compounds—the alicyclic ones) did not significantly influence *h*H_3_R affinities, with similar *K_i_* values obtained for analogs from all four subgroups (piperidine, 3- or 4-methylpiperidines and azepane). Among all the target compounds, 4-F substituted benzophenones with pentyloxyl linker presented the most promising activity, with low nanomolar *K_i_* values, i.e., 8 nM (lead **6**), 12 nM (**16**) and 13 nM (**30**). The H_3_R affinity of **6** was comparable with the reference pitolisant.

#### 2.3.2. Inhibition of *ee*AChE, *eq*BuChE, *h*AChE and *h*BuChE

All the target compounds were screened for their AChE and BuChE inhibitory activities, using Ellman’s colorimetric assay [41] and *electric eel* AChE (*ee*AChE) or *equine serum* BuChE (*eq*BuChE) enzymes. The screening concentration was 10 µM. For most active compounds, with at least 50% inhibitory potency at 10 µM, IC_50_ values were determined. Lead compound **6** was also tested using *human* AChE and *human* BuChE (*h*AChE and *h*BuChE) and its IC_50_ values for the inhibition of the human enzymes were determined. Tacrine was used as the reference. The results of the experiments are collected in Table 1. The inhibitory activity of most target structures toward *ee*AChE was good, with a micromolar range of the obtained IC_50_ values. However, all *ortho*-substituted benzophenones (**2**, **7**, **12**, **17**, **26**, **31**) and *para*-substituted **4** displayed lower inhibitory potency, with 3–41% of enzyme inhibition at screening concentration. Based on the performed assays, it was indicated that a six carbon atom linker was preferred for the *meta*-substituted benzophenones (e.g., **3** vs. **8**, or **13** vs. **18**, and **22** vs. **24**). Furthermore, the *ee*AChE inhibitory activity was more dependent on the presence of halogen substituents in the benzophenone scaffold, the position of the alkoxyl chain and the type of amine moieties than on small changes in the linker length. The most potent *ee*AChE inhibitor was found among azepane derivatives (**30**; IC_50_ = 1.110 µM). The piperidine-substituted lead **6** presented slightly weaker activity with IC_50_ = 2.303 µM and 9.59 µM for *ee*AChE and *h*AChE, respectively.

All the target compounds revealed significant activity toward *eq*BuChE, with IC_50_ values ranging from 161 nM (**35**) to 4.006 µM (**3**). In general, *para*-substituted benzophenones with a fluorine atom (4-F) were preferable. Their 4-Cl and unsubstituted analogs, as well as *meta*- and *ortho*-substituted derivatives, were less potent (**6** vs. **2**–**5**; **11** vs. **7**–**10**; **16** vs. **12**–**15**; **30** vs. **26**–**29** and **35** vs. **31**–**34**). The number of methylene groups present in the ether linkers was of less importance. Interestingly, some of the best *eq*BuChE inhibitors possessed five carbon atom linkers (e.g., **6**, **16**, **26** and **30**) and others, six carbon atom linkers (e.g., **31**, and **35**). Regarding the alicyclic amine moiety, compounds belonging to the azepane subgroup were the most promising *eq*BuChE inhibitors; almost all with nanomolar IC_50_ values. Compound **6** bearing the piperidine moiety, chosen to be the lead structure in terms of all selected AD targets, presented IC_50_ values of 172 nM and 1.16 µM for *eq*BuChE and *h*BuChE, respectively.

Compounds **2**, **7**, **12**, **17**, **26**, and **31** were found to be selective *eq*BuChE inhibitors. As both AChE and BuChE seem to be involved in acetylcholine hydrolysis, the non-selective cholinesterase inhibitory properties of most synthesized compounds are very valuable. However, those selective toward BuChE may be attractive because the BuChE activity increases continuously during AD progression at the expense of the declining activity of AChE [9].

Considering the aims of our research, all the synthesized molecules revealed good multidirectional activity toward H_3_R and cholinesterases, with the most interesting lead **6**.

#### 2.3.3. Kinetic Studies of *ee*AChE and *eq*BuChE Inhibition

For the most potent compound **6**, kinetic studies were performed to determine the mechanism of cholinesterase (*ee*AChE and *eq*BuChE) inhibition.

**Figure 3:**
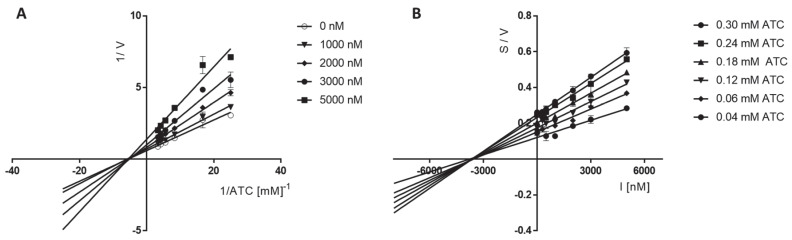
Lineweaver−Burk (**A**) and Cornish−Bowden (**B**) plots illustrating noncompetitive types of *ee*AChE inhibition by compound **6**. S = acetylthiocholine; V = initial velocity rate; I = inhibitor concentration.

**Figure 4:**
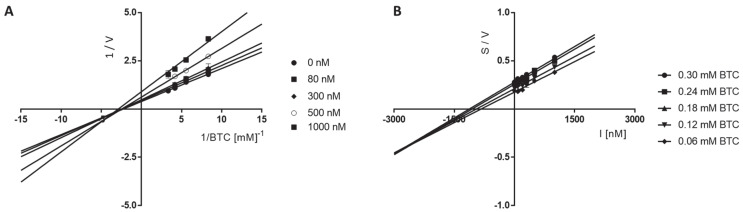
Lineweaver-Burk (**A**) and Cornish-Bowden (**B**) plots illustrating mixed types of *eq*BuChE inhibition by compound **6**. S = butyrylthiocholine; V = initial velocity rate; I = inhibitor concentration.

The Lineweaver–Burk plots obtained for **6** displayed a series of converging lines on the same point on the *x*-axis (1/[ATC] (Figure 3A)) or not much below the *x*-axis (1/[BTC] (Figure 4A)), profiling a non-competitive type mechanism of *ee*AChE and mix type mechanism of *eq*BuChE inhibition. For both *ee*AChE and *eq*BChE the plots showed increased slopes (decreased V_max_) at increasing concentrations of the inhibitor. Additionally, the preserved intercepts at the *x*-axis were observed for *ee*AChE (unchanged K_m_), whereas for *eq*BuChE different intercepts at the *x*-axis are presented in Figure 4A (increased K_m_). Noncompetitive types of *ee*AChE inhibition indicate an equal affinity of the inhibitor for the free enzyme and for the enzyme−substrate complex. On the other hand, mixed types of *eq*BuChE inhibition with increasing K_m_ at increasing concentrations of **6** indicate a higher affinity of the inhibitor to the free enzyme than to the enzyme−substrate complex.

The Cornish−Bowden plots (S/V vs. I) obtained for **6** displayed a series of converging lines on the same point on or below the *x*-axis for *ee*AChE or *eq*BuChE kinetic studies, respectively, (Figure 3 and Figure 4B) strengthening the noncompetitive (*ee*AChE) and mixed (*eq*BuChE) mechanism of cholinesterase inhibition.

#### 2.3.4. Human MAO B Inhibitory Activity

Inhibitory activity for *h*MAO B was evaluated in vitro in the fluorescence-based assay as described previously [32]. All compounds were tested at the concentration of 1 μM. Rasagiline and safinamide were used as the reference compounds. The results are collected in Table 1. None of the tested compounds showed inhibition for *h*MAO B higher than 50%, thus they were not further evaluated for both MAO B and MAO A inhibition and the IC_50_ values were not determined.

### 2.4. In Silico Docking Studies

#### 2.4.1. Molecular Modeling Studies to Histamine H_3_ Receptor

For this study, we used the previously described H_3_R homology model, constructed on the template of the crystal structure of the M_2_ muscarinic acetylcholine receptor (PDB ID: 3UON) [42].

**Figure 5:**
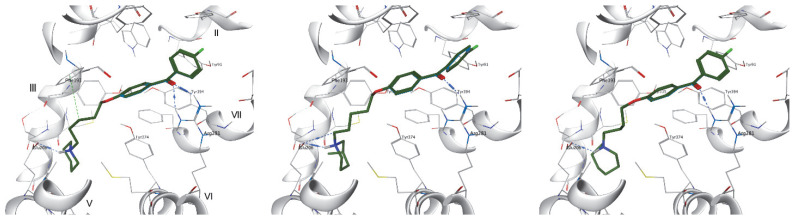
Putative binding modes of **6** (**left**), **16** (**middle**) and **30** (**right**) in histamine H_3_R binding site. Blue dashed lines express hydrogen bonds, green cation–π interactions; roman numbers denote TMs.

**Figure 6:**
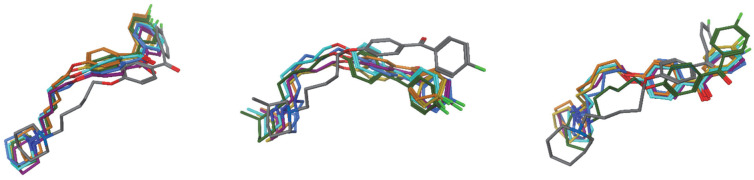
Orientations of **6** (**left**), **16** (**middle**) and **30** (**right**) during the MD simulations in 0 ps—grey, 100 ps—green, 200 ps—teal, 300 ps—green, 400 ps—violet, 500 ps—orange, 600 ps—yellow.

**Figure 7:**
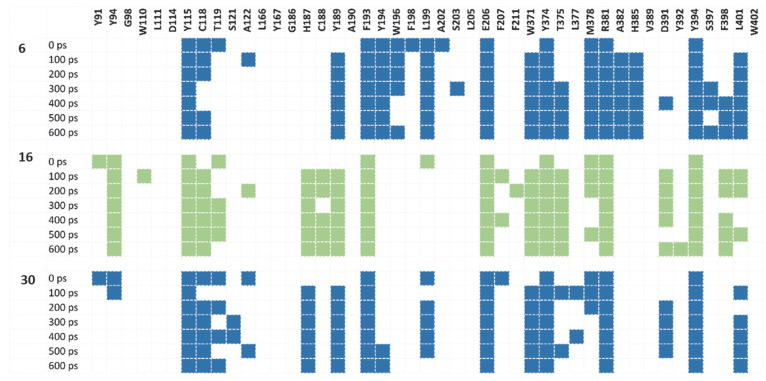
Interaction fingerprints of compounds **6**, **16** and **30** (blue, green and blue, respectively) and with H_3_R during MD simulation for particular frames. Colored box indicates any interaction between the ligand and the corresponding amino acid.

All the compounds were docked and characterized by relatively high docking score values and fit the H_3_R binding pocket. Independently of the alkyl chain length, west- and east-end variations, the docked ligands occupied the binding pocket in a similar mode preserving a crucial H_3_R antagonist/inverse agonist interactions, namely salt bridge and/or hydrogen bond formation between protonated amine nitrogen and Glu206^5.46^ (superscripts denote Ballesteros–Weinstein numbering)(Figure 5)[43]. The east-end benzophenone fragments occupied the space fenced by the aromatic features of Phe193, Tyr189 (ECL2) and Tyr 394^7.35^ on the sides and Tyr91^2.61^ and Tyr94^2.64^ on top. In the case of *ortho*-derivatives, the terminal benzene ring was placed perpendicular to Tyr189, stabilized by π-π stacking interactions and, due to its twisted position, a single hydrogen bond with one of the Arg381^6.58^ nitrogens. In the two remaining subgroups (*meta*- and *para*-) these rings were stabilized either by Tyr189 or/and Tyr394^7.35^, complimented by a double H-bond with the aforementioned arginine^6.58^. Additional stabilization through halogen bond formation with Tyr91^2.61^ for 4-Cl substituted derivatives was also present. The stability of the calculated poses for selected compounds (**6**, **16** and **30**) was further evaluated by means of short molecular dynamics (MD) simulations. From each simulation, seven poses were selected (starting pose, and after every 100 ps up to 600 ps). In most cases complexes appeared stable through the whole 600 ps simulation, retaining the key interactions, and the potential energy (U) of the atomic system at the level of ~1000 kcal/mol, with starting poses (marked in grey, Figure 6) similar to its orientation at the end of the simulation (marked yellow). However, a shift in the binding site with retained conformation appeared in the first 100 ps of the simulation. In the case of *ortho*-derivatives, a + 90° bend of benzophenone fragment occurred, which resulted in the change of hydrogen bond formation between the carbonyl group oxygen from Arg381^6.58^ to Tyr374^7.35^ and Tyr115^3.33^. Moreover, for the most potent 4-fluoro derivatives, a contact with highly conserved W371^6.48^ can be seen for six out of seven frames, as well as additional π-π stabilization for both benzene rings. Detailed analysis of the compound behavior during MD simulations is provided by examining the changes in their interactions with H_3_R (Figure 7). The results indicate a relatively consistent set of ligand–protein interactions that occur during the whole MD simulation. Most of the interactions occur within the TM3, TM5, TM6 and TM7 and ECL2, with consistent interactions with Tyr115^3.33^, Tyr189^45.51^, Glu206^5.46^, Tyr 374^6.51^, and Tyr394^7.35^.

#### 2.4.2. Analysis of Binding Mode within AChE

Docking studies of benzophenone derivatives allowed us to describe their binding modes in the AChE active site. The most active compounds were bound similarly: the heterocyclic rings interacted with the catalytic triad and anionic site residues. Next, linkers with the ether group were located near amino acids from the anionic site and acyl pocket, and the benzophenone aromatic fragment was close to the peripheral anionic site (PAS) residues [44].

**Figure 8:**
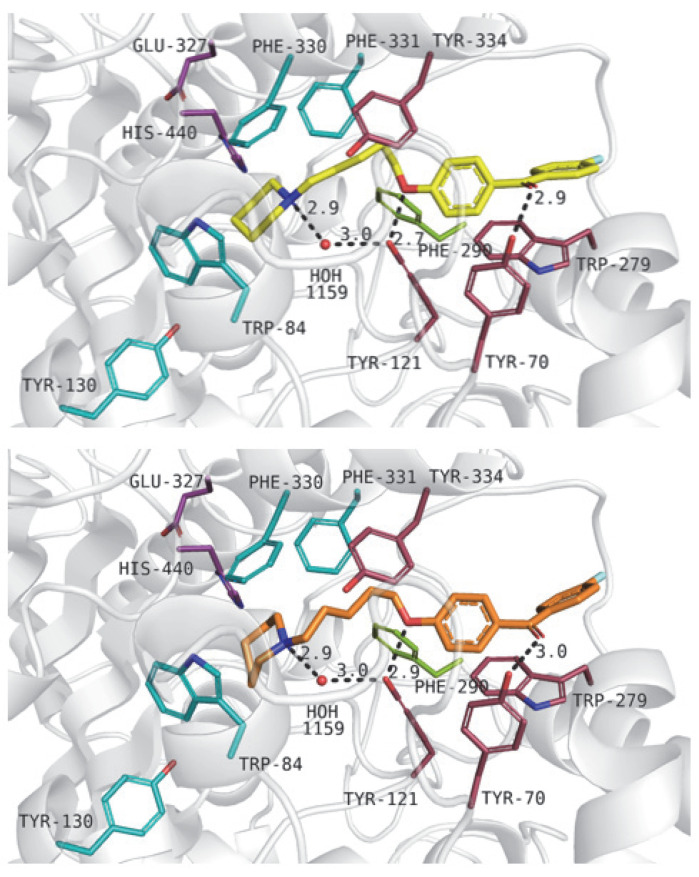
Binding modes of compound **30** (**upper panel**) and **6** (**lower panel**) within the acetylcholinesterase active site. Residues colored in purple—catalytic triad, blue—anionic site, pink—peripheral anionic site, green—acyl pocket.

Ligand **30** which showed the highest AChE inhibitory activity (IC_50_ = 1.11 µM) and compound **6** (IC_50_ = 2.306 µM) mimicked the binding mode of donepezil [45]. Figure 8 presents their best-ranked poses (inhibitor **30**—upper panel, **6**—lower panel).

The heterocyclic moieties of the above-mentioned compounds were close to His440 from the catalytic triad and Trp84, and Phe330 from the anionic site. The ionized amine groups from piperidine or azepane moieties interacted with aromatic residues through cation–π interactions. Protonated nitrogen atoms created a hydrogen bond with a water molecule (1159). Alkyl, flexible chains were responsible for hydrophobic interactions with Phe330, Phe331 from the anionic site and Phe290 from the acyl pocket of the enzyme. Additionally, ligands interacted with residues from the peripheral anionic site—Tyr334 and Tyr121. Oxygen atoms from alkyl linkers were able to create hydrogen bonds with a hydroxyl group of Tyr121 which next interacted with water 1159. This hydrogen bond network was described in our previous work [26]. Large, benzophenone fragments of the analyzed compounds were located between residues from PAS, such as Tyr121, Tyr70, Tyr334, and Trp279. Additionally, the oxygen atom from the carbonyl group interacted with Tyr70 through a hydrogen bond.

To check the stability of the pose obtained from docking studies, we decided to perform a molecular dynamics simulation. For calculations, we selected compound **6**. In the course of the MD simulation, we observed changes in the conformation of the ligand. During the last three ns of the simulation, the ligand revealed an RMSD value of 4.5 Å (Figure S1). Docking studies showed the presence of hydrogen bonds with hydroxy groups from Tyr121 and Tyr70. Interaction with Tyr70 was not found to be stable in the MD course; however, interaction with Tyr121 appeared again at the end of the simulation (Figure S2). Possibly, such behavior in the course of MD might be explained by the level of ligand activity (IC_50_ = 2.306 µM).

#### 2.4.3. Analysis of Binding Mode within BuChE

The results of docking to the butyrylcholinesterase active site were analyzed, and it was noticed that the most active ligands obtained similar binding modes to one another. The aliphatic rings with tertiary amine were found near the anionic site residues. Alkyl linkers interacted with residues from the peripheral anionic site. Aromatic rings were located between residues from the acyl pocket and the anionic site [44].

**Figure 9:**
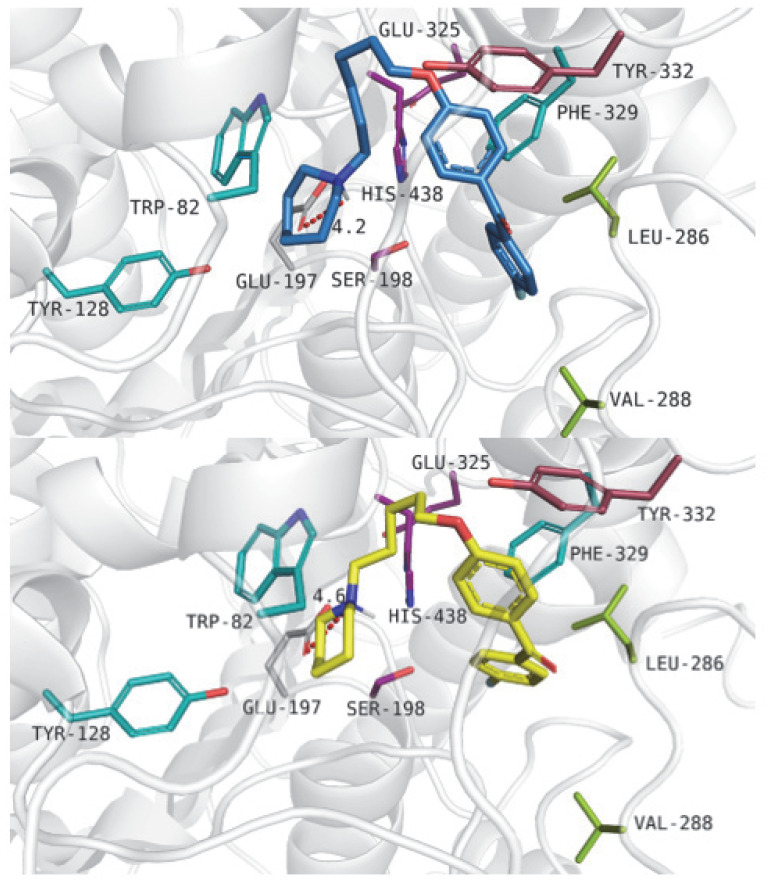
Binding mode of compound **35** (**upper panel**) and ligand **6** (**lower panel**) within the butyrylcholinesterase active site. Residues colored in purple—catalytic triad, blue—anionic site, pink—peripheral anionic site, green—acyl pocket.

Compounds **35** (IC_50_ = 0.161 µM) and **6** (IC_50_ = 0.172 µM) revealed binding modes as follows: azepane and piperidine fragments interacted with Tyr128 and Trp82 through cation–π interactions. Additionally, ionic interactions between the tertiary amine groups of the described compounds and the carboxyl group from Glu197 were observed. Alkyl chains were located near Tyr332 from the peripheral anionic site. Large, aromatic benzophenone fragments interacted with hydrophobic residues, such as Leu286 and Val288 from the acyl pocket and Phe329 counted to the anionic site. Figure 9 presents compounds **35** (upper panel) and **6** (lower panel).

**Figure 10:**
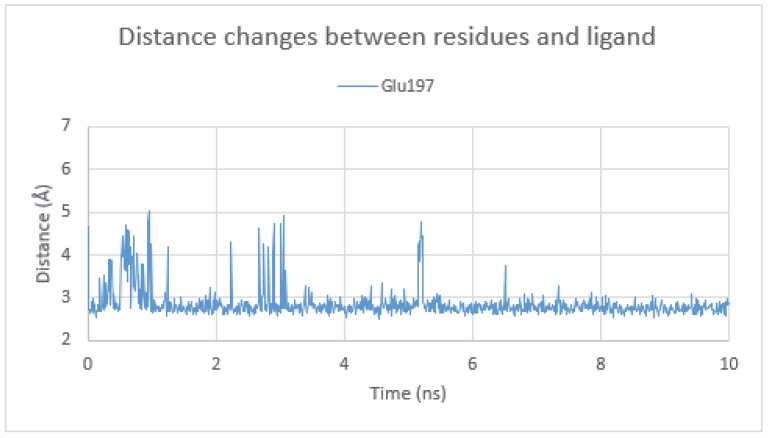
Distance changes between (N)Lig and (O)Glu197 in the BuChE active site during molecular dynamics simulation.

The binding mode of compound **6** within the butyrylcholinesterase active site was assessed by molecular dynamics simulation. Ligand presented a stable binding mode in the simulation and the RMSD value was at the level of 2 Å (Figure S3). These results are promising in comparison to the MD results for the same compound in a complex with AChE. Such behavior could be associated with higher biological activity at the nanomolar level (IC_50_ = 0.172 µM). Contrary to AChE, in the docking to BuChE, it was observed interaction between tertiary basic amine and the carboxy group of Glu197 which seems to be crucial for the ligand binding (Figure 10).

#### 2.4.4. Analysis of Binding Mode with Monoamine Oxidase B

The most active compounds showed convergent binding modes within the monoamine oxidase B active site. Heterocyclic rings with protonated nitrogen atoms were located in the aromatic pocket of the enzyme. Alkyl chains were able to create hydrophobic interactions with the protein. Large, aromatic benzophenone fragments were found near the cofactor (FAD).

**Figure 11:**
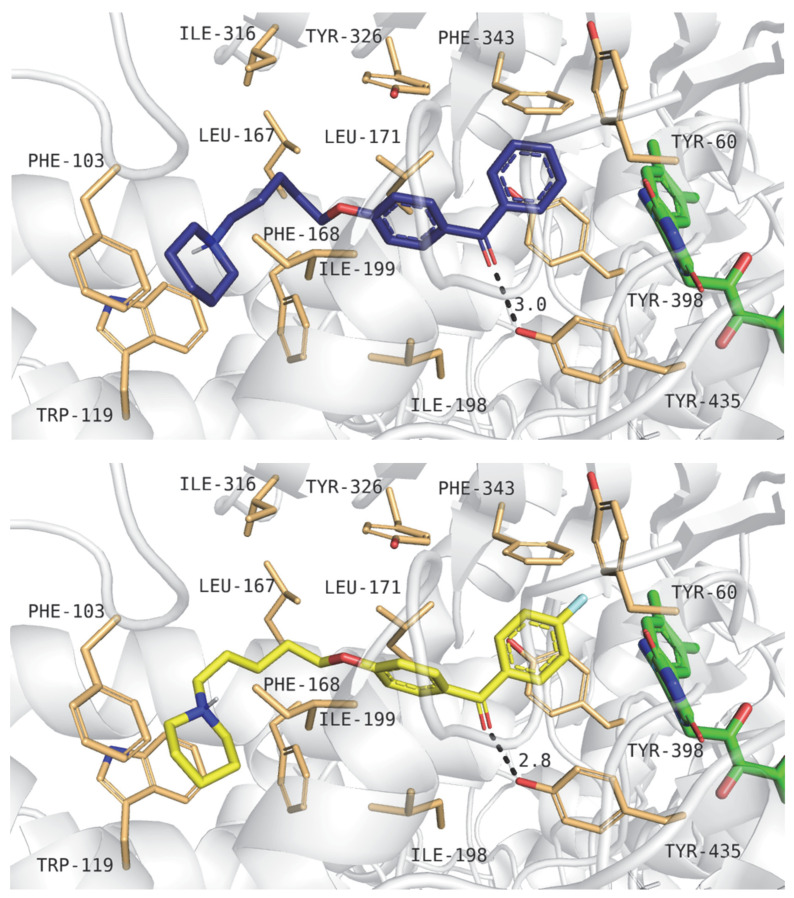
Binding mode of compounds **4** (**upper panel**) and **6** (**lower panel**) within the *human* MAO B active site. Amino acid residues are colored in sand, and cofactor FAD is in green.

Compound **4** was the most active in a screening concentration (% inh. at 1 µM = 44%). Figure 11 presents its binding mode (upper panel) and the binding mode of ligand **6** (lower panel; % inh at 1 µM = 25%). Piperidine moieties interacted with the following amino acids: Trp119, Phe103, Phe168 and Ile199. Tertiary amines from the piperidine rings created cation–π interactions with the Trp119 aromatic residue. Carbon linkers with ether groups were positioned near Leu167, Ile316, and Tyr326. The benzophenone fragments were located near FAD and interacted with Tyr398, Phe343, Tyr60 and Tyr435. It is worth noticing that the oxygen atoms from the carbonyl group could create a hydrogen bond with Tyr435.

Next, we performed an MD simulation for a complex of ligand **6** with the monoamine oxidase B. Analysis showed only small changes in the binding mode. The RMSD value of the ligand in the last two ns of the MD simulation was about 3 Å (Figure S4). A hydrogen bond with the hydroxy group of Tyr435 was present and it was noticed that additional interactions with the nitrogen atom of the amide group from Gln206 or weak interaction with the hydroxy group of Tyr398 appeared due to the residues and ligand movement (Figure S5).

### 2.5. Selected ADMET Properties

The chemical stability of the drug candidate affects its pharmacokinetic profile. Therefore, ADMET studies should be performed at the early stage of the drug development process. In vitro pharmacological profiling includes, among others, metabolic stability and hepatotoxicity. A metabolically unstable molecule may undergo rapid metabolism leading to the formation of metabolites that may not only be toxic but also devoid of biological activity. One of the possible harmful effects of compounds is liver damage. It is advisable to eliminate this risk at an early stage of preclinical development and to test the ability of the most promising compounds to cause hepatotoxicity. Another important factor for compounds expected to induce CNS effects is their ability to penetrate the BBB. On the one hand, the BBB plays an important role in protecting the brain, especially against harmful agents, but on the other hand, it can hinder the penetration of therapeutically important compounds. The evaluation of this ability of compounds at an initial stage may be a clue to the selection of suitable ligands for further pharmacological studies, especially in vivo studies. In neurodegenerative diseases, such as AD, the relevant neurons degenerate. If we can find drugs that can protect the nerves from damage, we have a chance to stop the progression of the disease. Therefore, it justifies testing the ability of compounds to protect neurons at an early stage of research.

In the presented study, in silico and in vitro methods were used to evaluate the selected properties of lead compound **6**. The metabolic stability was evaluated using in silico predictions (MetaSite 6.0.1) and in vitro experiments with mouse liver microsomes (MLMs). Additionally, the neuroprotection studies as well as the hepatotoxic effect of the most active compound **6** were evaluated with the use of the SH-SY5Y neuroblastoma cell line and hepatoma HepG2 cell line, respectively. Finally, the passive permeability through biological membranes for the lead **6** was also estimated.

#### 2.5.1. In Vitro Metabolic Stability of Compound **6**

**Figure 12:**
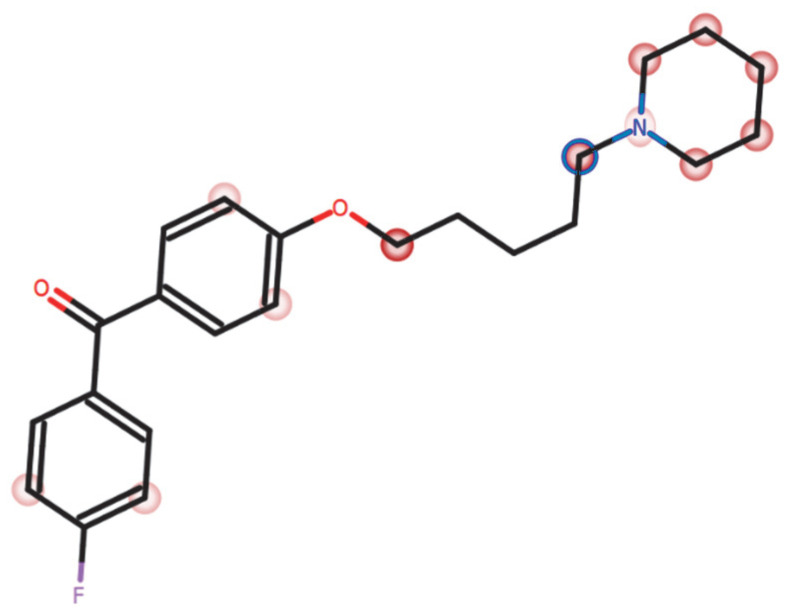
The MetaSite prediction of the most probable sites of metabolism for compound **6**. The sites with the highest probability of metabolic bioconversions are marked with blue circles while red marked circles indicate other potential sites of transformation (the darker the color, the higher the probability of metabolic degradation).

**Figure 13:**
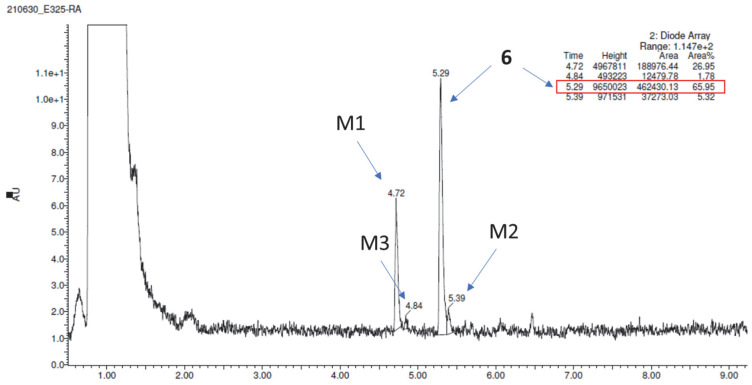
UPLC after 120 min incubation of compound **6** with MLMs.

The metabolic stability of **6** was investigated first in silico with the use of MetaSite 6.0.1 software [46]. The results showed that the piperidine moiety and the aliphatic linker were the most susceptible sites for metabolism (Figure 12). However, the *in silico* results were not completely confirmed in vitro. After incubation with MLMs for 120 min, the formation of three metabolites was observed (Figure 13) and none of them was obtained as modifications at the piperidine moiety. The molecular mass of the metabolite M1 (*m*/*z* = 372.39) and MS/MS ion fragmentation analysis suggested the reduction of the carbonyl group as the main metabolic pathway (Supporting information Figure S6; Table 2). In addition to the carbonyl group reduction, hydroxylation at two different sites of **6** was also identified (Supporting information Figure S6; Table 2). Moreover, the UPLC chromatograms showed that compound **6** was biotransformed at around 34%, whereas the metabolically unstable reference verapamil was at 76%. The obtained results illustrated the rather moderate metabolic stability of **6**.

#### 2.5.2. Neuroprotection Studies of Compound **6**

**Figure 14:**
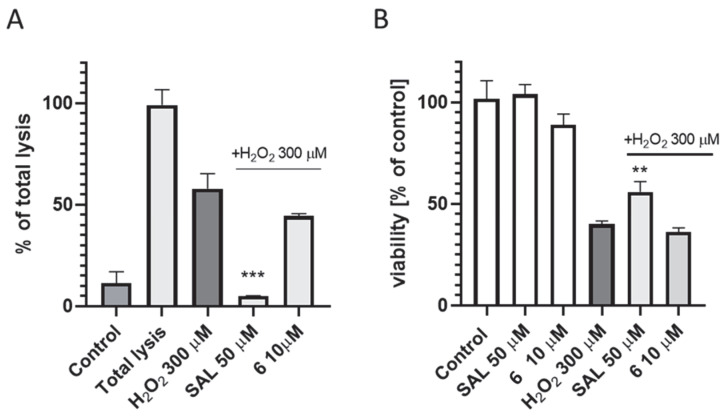
The effect of **6** at 10 µM and salsolinol (SAL; as the reference neuroprotectant) at 50 µM on SH-SY5Y *neuroblastoma* cells necrosis (**A**) and viability (**B**) damaged by 300 μM of H_2_O_2_ after 24 h of incubation. Statistical significance was set at *** *p* < 0.001, ** *p* < 0.01 by GraphPad Prism ^TM^ 8 software using One-way ANOVA and Bonferroni’s Multiple Comparison Post Test in comparison with the positive control H_2_O_2_ (300 μM).

The neuroprotection studies were performed with the use of the SH-SY5Y neuroblastoma cell line and H_2_O_2_ at a toxic concentration of 300 µM co-incubated with 10 µM (the highest non-toxic concentration, estimated before the assay) of **6** for 24 h. The cell necrosis and viability were next detected by LDH and MTS assays, respectively. In the LDH test slight inhibition of the necrosis process by **6** at a concentration of 10 µM was observed; however, it was statistically insignificant. At the same time salsolinol (SAL), the reference compound with previously confirmed neuroprotective activity [48], lowered the number of necrotic cells to the control level (Figure 14A). Statistical significance was calculated against the number of necrotic cells in the H_2_O_2_-treated population. Additionally, the MTS test was performed in which the viability of cells was measured. In this case, there was no increase in the viability of the SH-SY5Y cells population treated with H_2_O_2_ in the presence of **6** at 10 µM, whereas SAL also showed neuroprotective potential and statistically significantly increased the number of living cells compared to H_2_O_2_ alone (Figure 14B).

#### 2.5.3. The Permeability of Compound **6**

The PAMPA method was used to estimate the compound's passive permeability as described previously [32]. Caffeine was used as a well-permeable drug. Results presented in Table 3 show good permeability of compound **6** with a permeability coefficient value (*P_e_*) of 6.3 × 10^−6^ cm/s. Caffeine (*P_e_* = 15.1 × 10^−6^ cm/s) showed higher *P_e_* than **6** [49].

#### 2.5.4. Hepatotoxicity Studies of **6**

**Figure 15:**
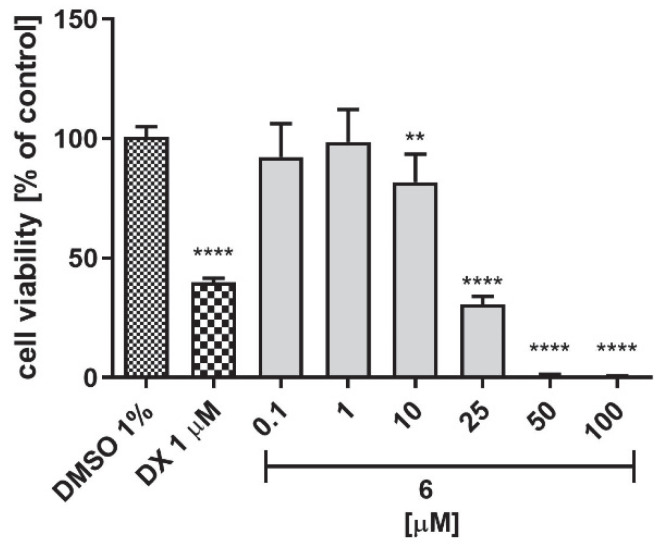
The influence of compound **6** and the reference cytostatic drug doxorubicin (DX) on the viability of hepatoma HepG2 cells after 72 h of incubation. DMSO was used as a control (1% in cell growth media). Statistical significance (**** *p* < 0.0001, ** *p* < 0.01) was analyzed by GraphPad Prism ^TM^ 6 software using a one-way ANOVA and Bonferroni’s multiple comparisons post-hoc test. The compounds were examined in quadruplicate.

There are many factors that can lead to liver damage, e.g., the simultaneous use of other drugs, genetic risk (polymorphism in CYP450), or personal factors (age, gender) [50]. The hepatotoxicity of a drug candidate is the main cause of the interruption of its further development, and in vitro toxicity testing is an essential part of drug safety testing at an early stage of its development. Thus, the hepatoma HepG2 cell line was used here to evaluate the hepatotoxicity of compound **6**. Doxorubicin (DX) at the concentration of 1 μM was used as the reference cytostatic drug. Compound **6** decreased cell viability starting from 10 μM concentration and above, while DX showed the toxic effect already at 1 μM concentration (Figure 15). Regardless, the obtained results indicate that the risk of hepatotoxicity cannot be excluded.

#### 2.5.5. In Silico Prediction of Mutagenic Effect of Selected Compounds

The Ames test is used worldwide as a first-order filter to assess the mutagenic potential of new chemicals and drugs. The calculations carried out for compound **6** and its analogs (i.e., compounds **16**: 3-methylpiperidine and **30**: azepane) were performed using the free online programme pkSCM (https://biosig.lab.uq.edu.au/pkcsm/prediction, accessed on 5 December 2022). In addition, the metabolites predicted by the MetaSite program for compound **6** and its analogs (i.e., compounds with a reduced carbonyl group; compounds with a hydroxyl group in the benzene and compounds with a reduced carbonyl and hydroxyl group in the benzene ring) were also added to this study. Calculations did not predict the Ames test activity for all compounds evaluated.

### 2.6. In Vivo Pharmacological Studies

Encouraged by both the BChE inhibitory activity and high H_3_R affinity, low hepatotoxicity, and proven passive permeability, we decided to test the effects of compound **6** on behavioral activities. Therefore, we investigated the in vivo activity of compound **6** in the Passive Avoidance Test (PAT) to evaluate its influence on memory and learning in mice with scopolamine-induced amnesia, which is widely referred to as a model simulating dementia in AD. Considering that the procognitive and analgesic effects are among many possible activities described for H_3_Rs ligands, we set out to investigate compound **6** in the formalin test to assess its analgesic potential.

**Figure 16:**
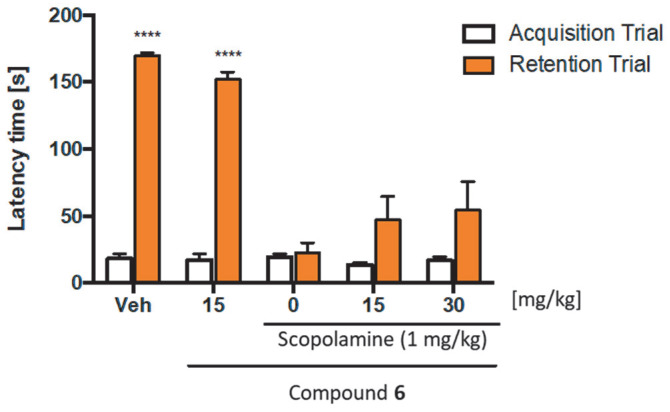
Activity of compound **6** in the Passive Avoidance Test. Compound **6** (15, or 30 mg/kg, i.p.) at the same volume of vehicle (1% Tween 80) was administered 30 min before the acquisition trial. Memory impairment was induced by scopolamine (1 mg/kg, s.c.) 20 min before the acquisition trial. 24 h after the acquisition trial, a retention trial was conducted for 180 s. Data represent means ± SEM (n = 8–10 per group); **** *p* < 0.0001 versus the scopolamine-treated group (0 on the *x*-axis). Statistical analysis: two-way ANOVA followed by Tukey’s Multiple Comparison Test.

In the PAT, mice with correct memory and learning processes behave contrary to their innate tendencies for preference of dark areas. The test consists of two trials, the acquisition and retention trials. In the first one, the mouse placed in the white compartment receives a mild foot shock when it innately crosses to the black compartment. During this phase, the animal learns that crossing into the dark compartment has negative consequences. In the next phase, the animal is again placed in the white compartment and the latency to escape from the white compartment is evaluated. The longer the latency, the better the memory performance. Thus, substances that impair memory, such as scopolamine, shorten the latency time in the retention trial, while substances improving memory reverse the scopolamine-induced impairment [51]. Our study showed (Figure 16) that compound **6** administrated at the dose of 15 mg/kg did not significantly reduce the latency time in the retention trial (169.80 ± 2.18 for vehicle-treated mice vs. 151.89 ± 5.46 for compound **6** treated mice). That proved that the tested compound itself did not induce memory impairment. It is worth emphasizing that in the retention trial, a dose-dependent slight increase in prolonged step-through latency was observed for compound **6** (15 and 30 mg/kg) in mice treated with scopolamine. Nevertheless, this compound did not statistically reverse the harmful effects of scopolamine (22.71 ± 7.56) at either the 15 mg/kg dose (47.07 ± 17.56) or the 30 mg/kg dose (51.56 ± 21.17). In summary, based on the above screening data in the PAT, it can be concluded that compound **6** has no significant harmful or beneficial effect on memory and learning.

**Figure 17:**
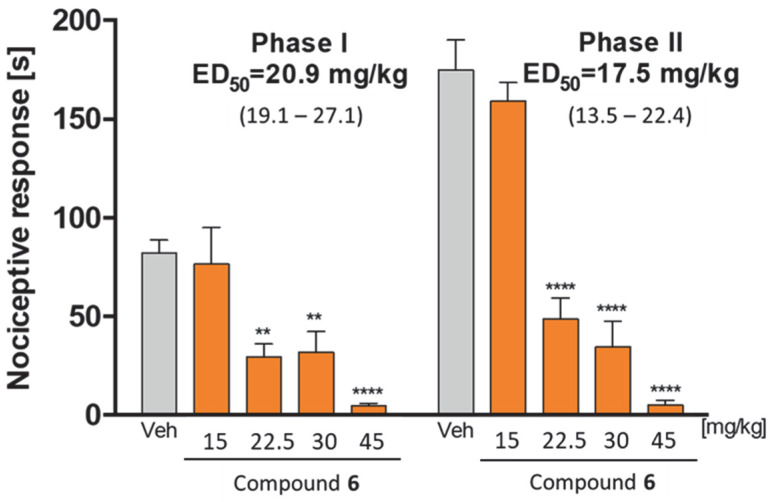
Antinociceptive activity of compound **6** in the formalin test. Results are shown as time of licking in phase I (0–5 min after intraplantar injection of formalin) and in phase II (15–30 min after formalin injection). Each value represents the mean ± S.E.M. for 8–10 animals. Statistical analysis: one-way ANOVA followed by post-hoc Dunnett’s test. Statistical significance compared to vehicle-treated animals (1% Tween 80): ** *p* < 0.01, **** *p* < 0.0001.

Continuing the preclinical characterization of compound **6**, we assessed its analgesic activity in the formalin test. Subcutaneous injection of formalin stimulates and then impairs sensory endings, which results in two distinct phases that can be observed during the test. Phase I mainly results from the immediate activation of nociceptors, while phase II results from the sensitization of spinal reflex circuits and peripherally developing inflammation. As it is presented in Figure 17, the i.p. administration of compound **6** prior to the subcutaneous injection of formalin significantly attenuated the nociceptive response in mice in both phases of the test. Its ED_50_ value in phase I was found to be 20.9 mg/kg, whereas the ED_50_ value in phase II was found to be 17.5 mg/kg. These results suggest significant analgesic properties of compound **6** in both acute (phase I) and inflammatory (phase II) pains.

## 3. Concluding Remarks

A series of 34 benzophenone derivatives was designed, synthesized, and evaluated in vitro as potential future AD multitarget therapeutics affecting the CNS neurotransmission. Compounds were divided into four subgroups, bearing piperidine, 3-methylpiperidine, 4-methylpiperidine, or the azepane moiety, linked by five to six carbon atom alkoxyl chains to the benzophenone scaffolds. Most of the compounds revealed significant biological activity toward the selected targets, i.e., human histamine H_3_ receptors and cholinesterase enzymes (AChE and BuChE). Compounds were also screened for human MAO B inhibitory potency, but none of them showed promising results (no more than 44% of enzyme inhibition at 1 µM). 4-F substituted benzophenones with pentyloxyl linkers were found to be the most potent *h*H_3_R antagonists/inverse agonists. Among them piperidine derivative **6** presented the highest *h*H_3_R affinity, being also a nonselective cholinesterase inhibitor (*h*H_3_R K_i_ = 8 nM; *ee*AChE IC_50_ = 2.31 µM; *h*AChE IC_50_ = 9.59 µM; *eq*BuChE IC_50_ = 0.17 µM; *h*BuChE IC_50_ = 1.16 µM; 25% of MAO B inhibition at 1 µM). In vitro studies of benzophenone derivatives were supplemented by molecular docking that allowed us to describe the binding modes of the compounds in the H_3_R, AChE, BuChE and MAO B active sites. The obtained binding modes of the lead compound **6** within H_3_R, AChE, BuChE and MAO B active sites were tested during molecular dynamics simulations. In further in vitro studies, compound **6** showed moderate metabolic stability in mouse liver microsomes (66% of the parent compound remained after 2 h of incubation), good permeability with a permeability coefficient value (P_e_) of 6.3 × 10^−6^ cm/s, and a lack of both hepatotoxicity effect in the 0.1–1 µM range in HepG2 cells, and unfortunately, neuroprotective activity in the LDH and MTS assays. Given the in vitro results, we selected compound **6** for in vivo evaluation of its memory-enhancing potency and its analgesic potential in mice. In the passive avoidance task, compound **6** used at a dose of 15 mg/kg and 30 mg/kg did not statistically prolong step-through latency in the model of scopolamine-induced cognitive dysfunction. On the other hand, compound **6** showed significant analgesic properties in both acute (ED_50_ = 20.9 mg/kg) and inflammatory (ED_50_ = 17.5 mg/kg) pain in the Formalin Test. The obtained results indicate a promising direction for further structural modifications of the herein-described compound **6**, considered a lead structure for further development.

## 4. Experimental Section

### 4.1. Synthesis

Reagents and solvents were obtained from commercial suppliers and used without further purification. Reactions were monitored by thin-layer chromatography using commercially available plates (Merck silica gel 60 F254 plates). The spots were visualized by UV lamp and Dragendorff’s reagent (solvent system: methylene chloride or methylene chloride: methanol 9:1). The structures and purity of compounds were confirmed by nuclear magnetic resonance (^1^H NMR and ^13^C NMR), mass spectra (LC/MS) and the elemental analysis (C, H, N). DMSO-*d*_6_ was used as a solvent for NMR spectra. Data were measured by Mercury 300 MHz PFG spectrometer (Varian, Palo Alto, CA, USA) or FT-NMR 500 MHz spectrometer (Joel Ltd., Akishima, Tokyo, Japan). The chemical shifts (*δ*) are reported in relation to tetramethylsilane (TMS) and the coupling constants (J) are expressed in Hz. The multiplicity of each peak is reported as: s, singlet; d, doublet; dd, doublet of doublets; t, triplet; quin, quintet; m, multiplet; br, broad. LC/MS spectra were performed on Waters TQ Detector Mass Spectrometer (Water Corporation, Milford, CT, USA). Retention times (t_R_) are given in minutes. The purity of compounds confirmed by UPLC/MS analysis was ≥95% (except **21**: 93.94% and **35**: 94.57%). The elemental analysis (C, H, N) for compounds was performed on Vario EL III Elemental Analyser (Hanau, Germany) and results agreed within 0.5% of the theoretical value (except **3**: 0.62% for H; **21**: 0.58% for C; **35**: 0.52% for C).

#### General Procedure for the Synthesis of Final Compounds

Compounds were synthesized and purified as described previously [29]. Briefly, a proper bromoalkoxybenzophenone (1 eq) was added to the solution of a proper amine (2 eq) in a mixture of ethanol and water (21:4, *v*/*v*) in the presence of K_2_CO_3_ (15 eq) and KI (the catalytic amount) and refluxed for 10–20 h. They were purified by extraction between CH_2_Cl_2_ and 1% HCl. The raw product was transformed into an oxalic acid salt in absolute ethanol and precipitated (if necessary) by adding diethyl-ether.

**Phenyl (2-((5-(piperidin-1-yl)pentyl)oxy)phenyl)methanone hydrogen oxalate (2)**

From (2-(5-Bromopentyloxy)phenyl)phenyl)methanone (0.87 g, 2.5 mmol) and piperidine (0.43 g, 5 mmol). Yield: 0.45 g (41%). Mp: 143–145 °C. C_23_H_29_NO_2_ × C_2_H_2_O_4_ (MW = 441.51). ^1^H NMR (500 MHz, DMSO-*d*_6_) δ: 7.56–7.68 (m, 3H), 7.41–7.54 (m, 3H), 7.32 (d, *J* = 6.30 Hz, 1H), 7.11 (d, *J* = 8.02 Hz, 1H), 7.05 (t, *J* = 7.45 Hz, 1H), 3.85 (t, *J* = 6.01 Hz, 2H), 2.98 (br. s., 4H), 2.59–2.74 (m, 2H), 1.67 (d, *J* = 5.16 Hz, 4H), 1.18–1.52 (m, 6H), 0.83 (quin, *J* = 7.30 Hz, 2H). ^13^C NMR (126 MHz, DMSO-*d*_6_) δ: 196.7, 165.4, 156.7, 138.3, 133.6, 132.8, 129.5, 129.5, 129.1, 129.0, 121.1, 113.2, 67.8, 56.2, 52.4, 28.3, 23.1, 23.0, 22.8, 22.0. LC-MS: purity 100% t_R_ = 5.04, (ESI) *m*/*z* [M + H]^+^ 352.27. Anal. calcd. for C_25_H_31_NO_6_: C, 68.00; H, 7.08; N, 3.17%. Found: C, 68.17; H, 7.16; N, 3.18%.

**Phenyl (3-((5-(piperidin-1-yl)pentyl)oxy)phenyl)methanone hydrogen oxalate (3)**

From (3-(5-bromopentyloxy)phenyl)phenyl)methanone (0.87g, 2.5 mmol) and piperidine (0.43 g, 5 mmol). Yield: 0.71 g (64%). Mp: 129–131 °C. C_23_H_29_NO_2_ × C_2_H_2_O_4_ (MW = 441.51). ^1^H NMR (300 MHz, DMSO-*d*_6_) δ: 7.68–7.76 (m, 2H), 7.66 (d, *J* = 7.44 Hz, 1H), 7.50–7.60 (m, 2H), 7.40–7.50 (m, 1H), 7.16–7.30 (m, 3H), 4.02 (t, *J* = 6.28 Hz, 2H), 2.82–3.20 (m, 6H), 1.60–1.83 (m, 8H), 1.34–1.57 (m, 4H). ^13^C NMR (126 MHz, DMSO-*d*_6_) δ: 196.1, 165.4, 159.1, 138.9, 137.5, 133.3, 130.3, 130.1, 129.1, 122.6, 119.6, 115.2, 67.9, 56.3, 52.4, 28.6, 23.4, 23.3, 23.0, 22.1. LC-MS: purity 99.37% t_R_ = 5.29, (ESI) *m*/*z* [M + H]^+^ 352.32. Anal. calcd. for C_26_H_3:3_NO_6_: C, 68.55; H, 7.30; N, 3.07%. Found: C, 68.04; H, 6.68; N, 3.15%.

**Phenyl (4-((5-(piperidin-1-yl)pentyl)oxy)phenyl)methanone hydrogen oxalate (4)**

From (4-(5-bromopentyloxy)phenyl)phenyl)methanone (1.73 g, 5 mmol) and piperidine (0.85 g, 10 mmol). Yield: 1.15 g (52%). Mp: 144–146 °C. C_23_H_29_NO_2_ × C_2_H_2_O_4_ (MW = 441.51). ^1^H NMR (500 MHz, DMSO-*d*_6_) δ: 7.70 (d, *J* = 8.59 Hz, 2H), 7.58–7.67 (m, 3H), 7.47–7.55 (m, 2H), 7.04 (d, *J* = 8.88 Hz, 2H), 4.05 (t, *J* = 6.44 Hz, 2H), 2.64–3.59 (m, 6H), 1.58–1.84 (m, 8H), 1.24–1.53 (m, 4H). ^13^C NMR (126 MHz, DMSO-*d*_6_) δ: 194.9, 165.4, 162.9, 138.3, 132.7, 132.6, 129.8, 129.0, 114.9, 68.1, 56.2, 52.4, 28.5, 23.4, 23.3, 23.0, 22.0. LC-MS: purity 100% t_R_ = 5.10, (ESI) *m*/*z* [M + H]^+^ 353.31. Anal. calcd. for C_25_H_31_NO_6_: C, 68.00; H, 7.08; N, 3.17%. Found: C, 67.81; H, 7.15; N, 3.17%.

**(4-Chlorophenyl) (4-((5-(piperidin-1-yl)pentyl)oxy)phenyl)methanone hydrogen oxalate (5)**

From (4-(5-bromopentyloxy)phenyl)(4-chlorophenyl)methanone (0.38 g, 1 mmol) and piperidine (0.17 g, 2 mmol) in the presence of K_2_CO_3_ (3 mmol) and KI (the catalytic amount). Yield: 0.17 g (35%). Mp: 138–140 °C. C_23_H_28_NO_2_Cl × C_2_H_2_O_4_ (MW = 475.96). ^1^H NMR (500 MHz, DMSO-*d*_6_) δ: 7.68 (dd, *J*_1_ = 8.59, *J*_2_ = 17.47 Hz, 4H), 7.52–7.62 (m, 2H), 7.05 (d, *J* = 8.59 Hz, 2H), 4.05 (t, *J* = 6.30 Hz, 2H), 2.60–3.64 (m, 6H), 1.60–1.80 (m, 8H), 1.30–1.54 (m, 4H). ^13^C NMR (101 MHz, DMSO-*d*_6_) δ: 193.7, 165.1, 163.0, 137.4, 136.9, 132.7, 131.6, 129.4, 129.0, 114.9, 68.1, 56.2, 52.4, 28.4, 23.4, 23.2, 23.0, 22.0. LC-MS: purity 99.57% t_R_ = 5.86, (ESI) *m*/*z* [M + H]^+^ 386.12. Anal. calcd. for C_25_H_30_NO_6_Cl: C, 63.08; H, 6.35; N, 2.94%. Found: C, 62.96; H, 6.36; N, 3.02%.

**(4-Fluorophenyl) (4-((5-(piperidin-1-yl)pentyl)oxy)phenyl)methanone hydrogen oxalate (6)**

From (4-(5-bromopentyloxy)phenyl)(4-fluorophenyl)methanone (0.36 g, 1 mmol) and piperidine (0.17 g, 2 mmol). Yield: 0.65 g (36%). Mp: 141–143 °C. C_23_H_28_NO_2_F × C_2_H_2_O_4_ (MW = 370.17). ^1^H NMR (500 MHz, DMSO-*d*_6_) δ: 7.62–7.79 (m, 4H), 7.35 (t, *J* = 8.88 Hz, 2H), 6.98–7.12 (m, 2H), 4.05 (t, *J* = 6.30 Hz, 2H), 2.63–3.48 (m, 6H), 1.59–1.83 (m, 8H), 1.29–1.56 (m, 4H). ^13^C NMR (101 MHz, DMSO-*d*_6_) δ: 193.5, 166.0, 165.1, 162.8, 134.8, 134.7, 132.6, 132.6, 132.5, 129.6, 116.1, 115.9, 114.8, 68.1, 56.2, 52.5, 28.4, 23.4, 23.2, 23.0, 22.0. LC-MS: purity 98.51% t_R_ = 5.32, (ESI) *m*/*z* [M + H]^+^ 384.20. Anal. calcd. for C_26_H_32_NO_6_F: C, 65.34; H, 6.58; N, 3.05%. Found: C, 65.45; H, 6.77; N, 3.00%.

**(2-((6-(Piperidin-1-yl)hexyl)oxy)phenyl)(phenyl)methanone hydrogen oxalate (7)**

From (2-(6-bromohexyloxy)phenyl)phenyl)methanone (0.9 g, 2.5 mmol) and piperidine (0.43 g, 5 mmol). Yield: 0.30 g (26%). Mp: 138–140 °C. C_24_H_31_NO_2_ × C_2_H_2_O_4_ (MW = 455.53). ^1^H NMR (400MHz, DMSO-*d*_6_) δ: 7.72–7.59 (m, 3 H), 7.59–7.41 (m, 3 H), 7.37 (dd, *J*_1_ = 1.2, *J*_2_ = 7.4 Hz, 1 H), 7.15 (d, *J* = 8.2 Hz, 1 H), 7.09 (t, *J* = 7.2 Hz, 1 H), 3.90 (t, *J* = 5.9 Hz, 2 H), 3.05 (br. s., 3 H), 2.83 (m, 2H), 1.83–1.62 (m, 4 H), 1.62–1.39 (m, 4 H), 1.34 (quin, *J* = 6.7 Hz, 2 H), 1.14–0.99 (m, 2 H), 0.98–0.84 (m, 2 H). ^13^C NMR (101 MHz, DMSO-*d*_6_) δ: 196.61, 165.10, 156.67, 138.22, 133.52, 132.71, 129.43, 129.39, 128.99, 128.96, 121.03, 113.10, 67.95, 56.27, 52.40, 28.56, 26.17, 25.05, 23.48, 23.00, 21.98. LC-MS: purity 100% t_R_ = 5.40, (ESI) *m*/*z* [M + H]^+^ 366.25. Anal. calcd. for C_26_H_33_NO_6_: C, 68.55; H, 7.30; N, 3.07%. Found: C, 68.46; H, 7.14; N, 3.07%.

**Phenyl (3-((6-(piperidin-1-yl)hexyl)oxy)phenyl)methanone hydrogen oxalate (8)**

From (3-(6-bromohexyloxy)phenyl)phenyl)methanone (1.81 g, 5 mmol) and piperidine (0.85 g, 10 mmol). Yield: 0.55 g (24%). Mp: 123–126 °C. C_24_H_31_NO_2_ × C_2_H_2_O_4_ (MW = 455.53). ^1^H NMR (500 MHz, DMSO-*d*_6_) δ: 7.70 (d, *J* = 7.16 Hz, 2H), 7.65 (t, *J* = 7.41 Hz, 1H), 7.53 (t, *J* = 7.73 Hz, 2H), 7.43 (t, *J* = 7.73 Hz, 1H), 7.14–7.25 (m, 3H), 3.98 (t, *J* = 6.30 Hz, 2H), 2.71–3.22 (m, 6H), 1.64–1.76 (m, 6H), 1.56–1.63 (m, 2H), 1.35–1.54 (m, 4H), 1.21–1.33 m, 2H). ^13^C NMR (126 MHz, DMSO-*d*_6_) δ: 196.1, 165.3, 159.1, 138.9, 137.5, 133.3, 130.3, 130.1, 129.1, 122.6, 119.6, 115.1, 68.1, 56.7, 54.0, 28.9, 26.6, 26.4, 25.6, 24.1, 23.5. LC-MS: purity 100% t_R_ = 5.65, (ESI) *m*/*z* [M + H]^+^ 366.34. Anal. calcd. for C_26_H_33_NO_6_: C, 68.55; H, 7.30; N, 3.07%. Found: C, 68.29; H, 7.70; N, 3.06%.

**Phenyl (4-((6-(piperidin-1-yl)hexyl)oxy)phenyl)methanone hydrogen oxalate (9)**

From (4-(6-bromohexyloxy)phenyl)phenyl)methanone (1.81 g, 5 mmol) and piperidine (0.85 g, 10 mmol). Yield: 0.69 g (30%). Mp: 133–136 °C. C_24_H_31_NO_2_ × C_2_H_2_O_4_ (MW = 455.53). ^1^H NMR (500 MHz, DMSO-*d*_6_) δ: 7.70 (d, *J* = 8.88 Hz, 2H), 7.58–7.66 (m, 3H), 7.46–7.56 (m, 2H), 7.04 (d, *J* = 8.88 Hz, 2H), 4.04 (t, *J* = 6.30 Hz, 2H), 2.66–3.28 (m, 6H), 1.57–1.84 (m, 8H), 1.36–1.55 (m, 4H), 1.18–1.35 (m, 2H). ^13^C NMR (126 MHz, DMSO-*d*_6_) δ: 194.9, 165.3, 163.0, 138.3, 132.7, 132.6, 129.8, 129.7, 129.0, 114.9, 68.3, 56.3, 52.4, 28.8, 26.4, 25.5, 23.6, 23.0, 22.0. LC-MS: purity 98.64% t_R_ = 5.45, (ESI) *m*/*z* [M + H]^+^ 367.33. Anal. calcd. for C_26_H_33_NO_6_: C, 68.55; H, 7.30; N, 3.07%. Found: C, 68.50; H, 7.37; N, 3.15%.

**(4-Chlorophenyl) (4-((6-(piperidin-1-yl)hexyl)oxy)phenyl)methanone hydrogen oxalate (10)**

From (4-(6-bromohexyloxy)phenyl)(4-chlorophenyl)methanone (0.49 g, 1.25 mmol) and piperidine (0.21 g, 2.5 mmol). Yield: 0.25 g (41%). Mp: 116–118 °C. C_24_H_30_NO_2_Cl × C_2_H_2_O_4_ (MW = 489.99). ^1^H NMR (400 MHz, DMSO-*d*_6_) δ: 7.72 (dd, *J*_1_ = 8.61, *J*_2_ = 11.74 Hz, 4H), 7.57–7.66 (m, 2H), 7.08 (d, *J* = 9.00 Hz, 2H), 4.08 (t, *J* = 6.26 Hz, 2H), 2.77–3.33 (m, 6H), 1.62–1.88 (m, 8H), 1.40–1.58 (m, 4H), 1.28–1.39 (m, 2H). ^13^C NMR (101 MHz, DMSO-*d*_6_) δ: 193.7, 165.2, 163.0, 137.4, 136.9, 132.7, 131.6, 129.4, 129.0, 114.9, 68.3, 56.3, 52.4, 28.7, 26.3, 25.5, 23.6, 23.0, 22.0. LC-MS: purity 100% t_R_ = 6.16, (ESI) *m*/*z* [M + H]^+^ 400,15. Anal. calcd. for C_26_H_32_NO_6_Cl: C, 63.73; H, 6.58; N, 2.86%. Found: C, 63.60; H, 6.57; N, 2.76%.

**(4-Fluorophenyl) (4-((6-(piperidin-1-yl)hexyl)oxy)phenyl)methanone hydrogen oxalate (11)**

From (4-(6-bromohexyloxy)phenyl)(4-fluorophenyl)methanone (0.38 g, 1 mmol) and piperidine (0.17 g, 2 mmol). Yield: 0.16 g (34%). Mp: 121–123 °C. C_24_H_30_NO_2_F × C_2_H_2_O_4_ (MW = 473.53). ^1^H NMR (400 MHz, DMSO-*d*_6_) δ: 7.69–7.85 (m, 4H), 7.38 (t, *J* = 8.80 Hz, 2H), 7.03–7.15 (m, 2H), 4.08 (t, *J* = 6.26 Hz, 2H), 3.08 (br. s., 6H), 1.61–1.84 (m, 8H), 1.40–1.58 (m, 4H), 1.30–1.40 (m, 2H). ^13^C NMR (101 MHz, DMSO-*d*_6_) δ: 193.5, 165.1, 162.9, 134.8, 132.6, 132.5, 129.6, 116.1, 115.9, 114.8, 68.3, 56.3, 52.4, 28.7, 26.3, 25.5, 23.6, 23.0, 22.0. LC-MS: purity 100% t_R_ = 5.67, (ESI) *m*/*z* [M + H]^+^ 384.35. Anal. calcd. for C_26_H_32_NO_6_F: C, 65.94; H, 6.81; N, 2.96%. Found: C, 65.97; H, 6.80; N, 2.95%.

**(2-((5-(3-Methylpiperidin-1-yl)pentyl)oxy)phenyl)(phenyl)methanone hydrogen oxalate (12)**

From (2-(5-bromopentyloxy)phenyl)phenyl)methanone (0.87 g, 2.5 mmol) and 3-methylpiperidine (0.50 g, 5 mmol)Yield: 0.50 g (44%). Mp: 147–150 °C. C_24_H_31_NO_2_ × C_2_H_2_O_4_ (MW = 455.53). ^1^H NMR (500 MHz, DMSO-*d*_6_) δ: 7.58–7.67 (m, 3H), 7.44–7.54 (m, 3H), 7.32 (dd, *J*_1_ = 1.72, *J*_2_ = 7.45 Hz, 1H), 7.11 (d, *J* = 8.02 Hz, 1H), 7.05 (t, *J* = 7.45 Hz, 1H), 3.86 (t, *J* = 6.01 Hz, 2H), 3.14–3.32 (m, 2H), 2.57–2.75 (m, 3H), 2.36 (t, *J* = 11.74 Hz, 1H), 1.80 (dd, *J*_1_ = 3.44, *J*_2_ = 6.30 Hz, 1H), 1.56–1.75 (m, 3H), 1.36–1.48 (m, 2H), 1.24–1.35 (m, 2H), 0.94–1.06 (m, 1H), 0.77–0.89 (m, 5H). ^13^C NMR (126 MHz, DMSO-*d*_6_) δ: 196.7, 165.3, 156.7, 138.3, 133.6, 132.8, 129.5, 129.5, 129.1, 129.0, 121.1, 113.2, 67.8, 57.8, 56.2, 51.9, 30.6, 29.0, 29.0, 28.9, 28.3, 23.1, 22.8, 22.7, 19.1.mLC-MS: purity 100% t_R_ = 5.40, (ESI) *m*/*z* [M + H]^+^ 366.29. Anal. Calcd. For C_26_H_33_NO_6_: C, 68.55; H, 7.30; N, 3.07%. Found: C, 68.78; H, 7.46; N, 3.11%.

**(3-((5-(3-Methylpiperidin-1-yl)pentyl)oxy)phenyl)(phenyl)methanone hydrogen oxalate (13)**

From (3-(5-bromopentyloxy)phenyl)phenyl)methanone (0.87 g, 2.5 mmol) and 3-methylpiperidine (0.50 g, 5 mmol). Yield: 0.73 g (64%). Mp: 112–115°C. C_24_H_31_NO_2_ × C_2_H_2_O_4_ (MW = 455.53). ^1^H NMR (300 MHz, DMSO-*d*_6_) δ: 7.61–7.77 (m, 3H), 7.50–7.60 (m, 2H), 7.40–7.50 (m, 1H), 7.17–7.28 (m, 3H), 4.02 (t, *J* = 6.15 Hz, 2H), 3.30 (t, *J* = 14.23 Hz, 2H), 2.87–3.02 (m, 2H), 2.67 (t, *J* = 11.03 Hz, 1H), 2.35–2.45 (m, 1H), 1.54–1.89 (m, 8H), 1.34–1.52 (m, 2H), 0.94–1.11 (m, 1H), 0.86 (d, *J* = 6.41 Hz, 3H). ^13^C NMR (126 MHz, DMSO-*d*_6_) δ: 196.1, 165.3, 159.1, 138.9, 137.5, 133.3, 130.3, 130.1, 129.1, 122.7, 119.6, 115.2, 67.9, 57.9, 56.3, 51.9, 30.6, 29.0, 29.0, 28.9, 28.6, 23.4, 23.3, 22.7, 19.1. LC-MS: purity 99.45% t_R_ = 5.61, (ESI) *m*/*z* [M + H]^+^ 366.35. Anal. Calcd. For C_26_H_33_NO_6_: C, 68.55; H, 7.30; N, 3.07%. Found: C, 68.45; H, 7.14; N, 3.09%.

**(4-((5-(3-Methylpiperidin-1-yl)pentyl)oxy)phenyl)(phenyl)methanone hydrogen oxalate (14)**

From (4-(5-bromopentyloxy)phenyl)phenyl)methanone (1.73 g, 5 mmol) and 3-methylpiperidine (0.99 g, 10 mmol). Yield: 1.10 g (48%). Mp: 115–118 °C. C_24_H_31_NO_2_ × C_2_H_2_O_4_ (MW = 455.53). ^1^H NMR (500 MHz, DMSO-*d*_6_) δ: 7.70 (d, *J* = 8.88 Hz, 2H), 7.58–7.67 (m, 3H), 7.46–7.55 (m, 2H), 7.04 (d, *J* = 8.88 Hz, 2H), 4.05 (t, *J* = 6.30 Hz, 2H), 3.21–3.45 (m, 2H), 2.94 (t, *J* = 7.59 Hz, 2H), 2.66 (t, *J* = 10.88 Hz, 1H), 2.27–2.43 (m, 1H), 1.57–1.92 (m, 8H), 1.40 (quin, *J* = 7.52 Hz, 2H), 0.95–1.10 (m, 1H), 0.84 (d, *J* = 6.59 Hz, 3H). ^13^C NMR (126 MHz, DMSO-*d*_6_) δ: 194.9, 165.3, 162.9, 138.3, 132.7, 132.6, 129.8, 129.0, 114.9, 68.1, 57.9, 51.9, 30.6, 28.5, 23.4, 23.3, 19.1. LC-MS: purity 100% t_R_ = 5.41, (ESI) *m*/*z* [M + H]^+^ 366.38. Anal. Calcd. For C_26_H_33_NO_6_: C, 68.55; H, 7.30; N, 3.07%. Found: C, 68.50; H, 7.40; N, 3.04%.

**(4-Chlorophenyl) (4-((5-(3-methylpiperin-1-yl)pentyl)oxy)phenyl)methanone hydrogen oxalate (15)**

From (4-(5-bromopentyloxy)phenyl)(4-chlorophenyl)methanone (0.38 g, 1 mmol) and 3-methylpiperidine. Yield: 0.19 g (39%). Mp: 149–151 °C. C_24_H_30_NO_2_Cl × C_2_H_2_O_4_ (MW = 489.99). ^1^H NMR (400 MHz, DMSO-*d*_6_) δ: 7.68–7.81 (m, 4H), 7.56–7.66 (m, 2H), 7.09 (d, *J* = 9.00 Hz, 2H), 4.10 (t, *J* = 6.26 Hz, 2H), 3.26–3.46 (m, 2H), 2.89–3.06 (m, 2H), 2.63–2.77 (m, 1H), 2.45 (t, *J* = 11.74 Hz, 1H), 1.60–1.96 (m, 8H), 1.37–1.52 (m, 2H), 0.99–1.15 (m, 1H), 0.89 (d, *J* = 6.65 Hz, 3H). ^13^C NMR (101 MHz, DMSO-*d*_6_) δ: 193.7, 165.1, 163.0, 137.4, 136.9, 132.7, 131.6, 129.4, 129.0, 114.9, 68.1, 57.9, 52.0, 30.6, 28.4, 23.4, 23.2, 19.1. LC-MS: purity 100% t_R_ = 6.15, (ESI) *m*/*z* [M + H]^+^ 400.15. Anal. Calcd. For C_26_H_32_NO_6_Cl: C, 63.73; H, 6.58; N, 2.86%. Found: C, 63.40; H, 6.39; N, 2.92%.

**(4-Fluorophenyl) (4-((5-(3-methylpiperidin-1-yl)pentyl)oxy)phenyl)methanone hydrogen oxalate (16)**

From (4-(5-bromopentyloxy)phenyl)(4-fluorophenyl)methanone (0.36 g, 1 mmol) and 3-methylpiperidine (0.20 g, 2 mmol). Yield: 0.17 g (36%). Mp: 123–126 °C. C_24_H_30_NO_2_F × C_2_H_2_O_4_ (MW = 473.53). ^1^H NMR (400 MHz, DMSO-*d*_6_) δ: 7.67–7.84 (m, 4H), 7.38 (t, *J* = 8.80 Hz, 2H), 7.09 (d, *J* = 9.00 Hz, 2H), 4.09 (t, *J* = 6.26 Hz, 2H), 3.22–3.46 (m, 2H), 2.89–3.05 (m, 2H), 2.70 (t, *J* = 10.17 Hz, 1H), 2.45 (t, *J* = 11.74 Hz, 1H), 1.61–1.95 (m, 8H), 1.36–1.55 (m, 2H), 0.97–1.13 (m, 1H), 0.79–0.94 (m, 3H). ^13^C NMR (101 MHz, DMSO-*d*_6_) δ: 193.5, 166.0, 165.2, 163.5, 162.9, 134.8, 134.7, 132.6, 132.6, 132.5, 129.6, 116.1, 115.9, 114.8, 68.1, 57.9, 56.2, 51.9, 30.6, 28.9, 28.4, 23.4, 23.2, 22.7, 19.1. LC-MS: purity 95.44% t_R_ = 5.53, (ESI) *m*/*z* [M + H]^+^ 384.20. Anal. Calcd. For C_26_H_32_NO_6_F: C, 65.94; H, 6.81; N, 2.96%. Found: C, 66.04; H, 6.96; N, 2.96%.

**(2-((6-(3-Methylpiperidin-1-yl)hexyl)oxy)phenyl)(phenyl)methanone hydrogen oxalate (17)**

From (2-(6-bromohexyloxy)phenyl)phenyl)methanone (0.9 g, 2.5 mmol) and 3-methylpiperidine (0.5 g, 5 mmol). After crystallization from C_2_H_5_OH. Yield: 0.23 g (25%). Mp: 116–118 °C. C_25_H_33_NO_2_ × C_2_H_2_O_4_ (MW = 469.57). ^1^H NMR (400 MHz, DMSO-*d*_6_) δ: 7.60–7.71 (m, 3H), 7.47–7.57 (m, 3H), 7.36 (dd, *J*_1_ = 1.56, *J*_2_ = 7.43 Hz, 1H), 7.15 (d, *J* = 8.22 Hz, 1H), 7.09 (t, *J* = 7.24 Hz, 1H), 3.89 (t, *J* = 6.06 Hz, 2H), 3.20–3.39 (m, 2H), 2.78–2.89 (m, 2H), 2.61–2.74 (m, 1H), 2.42 (t, *J* = 11.74 Hz, 1H), 1.82–1.95 (m, 1H), 1.63–1.81 (m, 3H), 1.46 (td, *J* = 7.78, 15.36 Hz, 2H), 1.28–1.39 (m, 2H), 0.97–1.14 (m, 3H), 0.86–0.97 (m, 5H). ^13^C NMR (101 MHz, DMSO-*d*_6_) δ: 196.6, 165.2, 156.7, 138.2, 133.5, 132.7, 129.4, 129.4, 129.0, 121.0, 113.1, 68.0, 57.8, 56.3, 51.8, 30.5, 28.9, 28.6, 26.2, 25.1, 23.5, 22.6, 19.1. LC-MS: purity 100% t_R_ = 5.66, (ESI) *m*/*z* [M + H]^+^ 380.48. Anal. calcd. for C_27_H_35_NO_6_: C, 69.06; H, 7.51; N, 2.98%. Found: C, 68.86; H, 7.67; N, 2.85%.

**(3-((6-(3-Methylpiperidin-1-yl)hexyl)oxy)phenyl)(phenyl)methanone hydrogen oxalate (18)**

From (3-(6-bromohexyloxy)phenyl)phenyl)methanone (1.81 g, 5 mmol) and 3-methylpiperidine (0.99 g, 10 mmol). Yield: 0.63 g (27%). Mp: 121–124 °C. C_25_H_33_NO_2_ × C_2_H_2_O_4_ (MW = 469.57). ^1^H NMR (500 MHz, DMSO-*d*_6_) δ: 7.68–7.73 (m, 2H), 7.61–7.67 (m, 1H), 7.49–7.57 (m, 2H), 7.39–7.46 (m, 1H), 7.15–7.24 (m, 3H), 3.98 (t, *J* = 6.44 Hz, 2H), 3.19–3.37 (m, 2H), 2.90 (t, *J* = 7.73 Hz, 2H), 2.65 (t, *J* = 10.88 Hz, 1H), 2.39 (t, *J* = 11.89 Hz, 1H), 1.75–1.89 (m, 1H), 1.55–1.74 (m, 7H), 1.37–1.47 (m, 2H), 1.18–1.34 (m, 2H), 0.93–1.06 (m, 1H), 0.80–0.88 (m, 3H). ^13^C NMR (126 MHz, DMSO-*d*_6_) δ: 196.1, 165.2, 159.1, 138.9, 137.5, 133.3, 130.3, 130.1, 129.1, 122.6, 119.6, 115.2, 68.1, 56.4, 52.5, 28.9, 26.4, 25.6, 23.6, 23.1, 22.0. LC-MS: purity 100% t_R_ = 5.99, (ESI) *m*/*z* [M + H]^+^ 380.23. Anal. calcd. for C_27_H_35_NO_6_: C, 69.06; H, 7.51; N, 2.98%. Found: C, 68.87; H, 7.65; N, 3.01%.

**(4-((6-(3-Methylpiperidin-1-yl)hexyl)oxy)phenyl)(phenyl)methanone hydrogen oxalate (19)**

From (4-(6-bromohexyloxy)phenyl)phenyl)methanone (1.81 g, 5 mmol) and 3-methylpiperidine (0.99 g, 10 mmol). Yield: 0.73 g (31%). Mp: 93–96 °C. C_25_H_33_NO_2_ × C_2_H_2_O_4_ (MW = 469.56). ^1^H NMR (500 MHz, DMSO-*d*_6_) δ: 7.70 (d, *J* = 8.59 Hz, 2H), 7.58–7.67 (m, 3H), 7.47–7.55 (m, 2H), 7.04 (d, *J* = 8.59 Hz, 2H), 4.03 (t, *J* = 6.44 Hz, 2H), 3.20–3.37 (m, 2H), 2.90 (t, *J* = 7.88 Hz, 2H), 2.65 (t, *J* = 10.60 Hz, 1H), 2.39 (t, *J* = 11.60 Hz, 1H), 1.77–1.89 (m, 1H), 1.55–1.76 (m, 7H), 1.36–1.49 (m, 2H), 1.20–1.35 (m, 2H), 0.93–1.07 (m, 1H), 0.84 (d, *J* = 6.59 Hz, 3H). ^13^C NMR (101 MHz, DMSO-*d*_6_) δ: 194.93, 165.36, 162.96, 138.32, 132.74, 132.61, 129.76, 129.73, 128.99, 114.85, 68.30, 57.87, 51.92, 30.63, 28.77, 26.40, 25.53, 23.63, 19.13. LC-MS: purity 95.73% t_R_ = 5.75, (ESI) *m*/*z* [M + H]^+^ 381.35. Anal. calcd. for C_27_H_35_NO_6_: C, 69.06; H, 7.51; N, 2.98%. Found: C, 69.49; H, 7.75; N, 3.16%.

**(4-Chlorophenyl) (4-((6-(3-methylpiperin-1-yl)hexyl)oxy)phenyl)methanone hydrogen oxalate (20)**

From (4-(6-bromohexyloxy)phenyl)(4-chlorophenyl)methanone (0.39 g, 1 mmol) and 3-methylpiperidine (0.20 g, 2 mmol). Yield: 0.19 g (38%). Mp: 132–135 °C. C_25_H_32_NO_2_Cl × C_2_H_2_O_4_ (MW = 504.01). ^1^H NMR (400 MHz, DMSO-*d*_6_) δ: 7.66–7.79 (m, 4H), 7.55–7.65 (m, 2H), 7.09 (d, *J* = 9.00 Hz, 2H), 4.08 (t, *J* = 6.46 Hz, 2H), 3.22–3.47 (m, 2H), 2.86–3.04 (m, 2H), 2.63–2.79 (m, 1H), 2.44 (t, *J* = 11.74 Hz, 1H), 1.60–1.96 (m, 8H), 1.45 (quin, *J* = 7.34 Hz, 2H), 1.27–1.39 (m, 2H), 0.98–1.14 (m, 1H), 0.89 (d, *J* = 6.26 Hz, 3H). ^13^C NMR (101 MHz, DMSO-*d*_6_) δ: 193.7, 165.1, 163.0, 137.4, 136.9, 132.7, 131.6, 129.4, 129.0, 114.9, 68.3, 57.8, 56.3, 51.9, 30.6, 28.9, 28.7, 26.3, 25.5, 23.6, 22.6, 19.1. LC-MS: purity 100% t_R_ = 6.41, (ESI) *m*/*z* [M + H]^+^ 414.17. Anal. calcd. for C_27_H_34_NO_6_Cl: C, 64.34; H, 6.80; N, 2.78%. Found: C, 64.35; H, 6.88; N, 2.70%.

**(4-Fluorophenyl) (4-(6-(3-methylpiperin-1-yl)hexyloxy)phenyl)methanone hydrogen oxalate (21)**

From (4-(6-bromohexyloxy)phenyl)(4-chlorophenyl)methanone (0.38 g, 1 mmol) and 3-methylpiperidine (0.20 g, 2 mmol). Yield: 0.12 g (24%). Mp: 105–108 °C. C_25_H_32_NO_2_F × C_2_H_2_O_4_ (MW = 487.55). ^1^H NMR (400 MHz, DMSO-*d*_6_) δ: 7.67–7.87 (m, 4H), 7.38 (t, *J* = 8.80 Hz, 2H), 7.08 (d, *J* = 8.61 Hz, 2H), 4.08 (t, *J* = 6.46 Hz, 2H), 3.24–3.42 (m, 2H), 2.86–3.04 (m, 2H), 2.69 (t, *J* = 10.37 Hz, 1H), 2.43 (t, *J* = 11.74 Hz, 1H), 1.58–1.95 (m, 8H), 1.46 (quin, *J* = 7.24 Hz, 2H), 1.28–1.38 (m, 2H), 0.96–1.13 (m, 1H), 0.79–0.95 (m, 3H). ^13^C NMR (101 MHz, DMSO-*d*_6_) δ: 193.5, 166.0, 165.1, 163.5, 162.9, 134.8, 134.7, 132.6, 132.5, 129.6, 116.1, 115.9, 114.8, 68.3, 57.9, 56.4, 51.9, 30.6, 29.0, 28.7, 26.3, 25.5, 23.6, 22.7, 19.1. LC-MS: purity 93.94% t_R_ = 5.86, (ESI) *m*/*z* [M + H]^+^ 398.22. Anal. calcd. for C_27_H_34_NO_6_F: C, 66.51; H, 7.03; N, 2.87%. Found: C, 67.09; H, 7.02; N, 2.95%.

**(3-((5-(4-Methylpiperidin-1-yl)pentyl)oxy)phenyl)(phenyl)methanone hydrogen oxalate (22)**

From (3-(5-bromopentyloxy)phenyl)phenyl)methanone (0.87 g, 2.5 mmol) and 4-methylpiperidine (0.50 g, 5 mmol). Yield: 0.47 g (41%). Mp: 166–168 °C. C_24_H_31_NO_2_ × C_2_H_2_O_4_ (MW = 455.53). ^1^H NMR (300 MHz, DMSO-*d*_6_) δ: 7.72 (d, *J* = 6.92 Hz, 2H), 7.66 (d, *J* = 7.44 Hz, 1H), 7.50–7.60 (m, 2H), 7.40–7.49 (m, 1H), 7.18–7.28 (m, 3H), 4.02 (t, *J* = 6.28 Hz, 2H), 3.34 (d, *J* = 12.31 Hz, 2H), 2.90–3.01 (m, 2H), 2.80 (t, *J* = 11.41 Hz, 2H), 1.50–1.81 (m, 7H), 1.25–1.49 (m, 4H), 0.89 (d, *J* = 6.41 Hz, 3H). ^13^C NMR (126 MHz, DMSO-*d*_6_) δ: 196.1, 165.2, 159.1, 138.9, 137.5, 133.3, 130.3, 130.1, 129.1, 122.7, 119.6, 115.2, 67.9, 56.5, 52.0, 28.6, 23.6, 23.3, 21.4, 21.4, 19.1. LC-MS: purity 98.81% t_R_ = 5.59, (ESI) *m*/*z* [M + H]^+^ 366.35. Anal. calcd. for C_26_H_33_NO_6_: C, 68.55; H, 7.30; N, 3.07%. Found: C, 68.40; H, 7.08; N, 3.06%.

**(4-((5-(4-Methylpiperidin-1-yl)pentyl)oxy)phenyl)(phenyl)methanone hydrogen oxalate (23)**

From (4-(5-bromopentyloxy)phenyl)phenyl)methanone (1.73 g, 5 mmol) and 4-methylpiperidine (0.99 g, 10 mmol). Yield: 1.10 g (48%). Mp: 148–150 °C. C_24_H_31_NO_2_ × C_2_H_2_O_4_ (MW = 455.53). ^1^H NMR (500 MHz, DMSO-*d*_6_) δ: 7.70 (d, *J* = 8.88 Hz, 2H), 7.58–7.67 (m, 3H), 7.47–7.55 (m, 2H), 7.04 (d, *J* = 8.88 Hz, 2H), 4.05 (t, *J* = 6.30 Hz, 2H), 3.33 (d, *J* = 10.31 Hz, 2H), 2.87–3.02 (m, 2H), 2.79 (br. s., 2H), 1.62–1.83 (m, 6H), 1.55 (br. s., 1H), 1.24–1.45 (m, 4H), 0.82–0.93 (m, 3H). ^13^C NMR (126 MHz, DMSO-*d*_6_) δ: 194.9, 165.4, 162.9, 138.3, 132.7, 132.6, 129.8, 129.0, 114.9, 68.1, 55.9, 51.9, 31.0, 28.5, 23.5, 23.3, 21.4. LC-MS: purity 100% t_R_ = 5.39, (ESI) *m*/*z* [M + H]^+^ 366.38. Anal. calcd. for C_26_H_33_NO_6_: C, 68.55; H, 7.30; N, 3.07%. Found: C, 68.24; H, 7.35; N, 3.07%.

**(3-((6-(4-Methylpiperidin-1-yl)hexyl)oxy)phenyl)(phenyl)methanone hydrogen oxalate (24)**

From (3-(6-bromohexyloxy)phenyl)phenyl)methanone (1.81 g, 5 mmol) and 4-methylpiperidine (0.99 g, 10 mmol). Yield: 0.33 g (14%). Mp: 120–123 °C. C_25_H_33_NO_2_ × C_2_H_2_O_4_ (MW = 469.57). ^1^H NMR (500 MHz, DMSO-*d*_6_) δ: 7.67–7.73 (m, 2H), 7.61–7.67 (m, 1H), 7.49–7.57 (m, 2H), 7.39–7.46 (m, 1H), 7.16–7.26 (m, 3H), 3.98 (t, *J* = 6.30 Hz, 2H), 3.22–3.39 (m, 2H), 2.85–2.98 (m, 2H), 2.77 (t, *J* = 11.03 Hz, 2H), 1.65–1.78 (m, 4H), 1.49–1.64 (m, 3H), 1.19–1.46 (m, 6H), 0.82–0.93 (m, 3H). ^13^C NMR (126 MHz, DMSO-*d*_6_) δ: 196.1, 165.3, 159.1, 138.9, 137.5, 133.3, 130.3, 130.1, 129.1, 122.6, 119.6, 115.2, 68.1, 28.9, 26.4, 25.6, 23.8. LC-MS: purity 100% t_R_ = 5.64, (ESI) *m*/*z* [M + H]^+^ 380.10. Anal. calcd. for C_27_H_35_NO_6_: C, 69.06; H, 7.51; N, 2.98%. Found: C, 68.82; H, 7.58; N, 2.97%.

**(4-((6-(4-Methylpiperidin-1-yl)hexyl)oxy)phenyl)(phenyl)methanone hydrogen oxalate (25)**

From (4-(6-bromohexyloxy)phenyl)phenyl)methanone (1.81 g, 5 mmol) and 4-methylpiperidine (0.99 g, 10 mmol). Yield: 1.03 g (44%). Mp: 139–142 °C. C_25_H_33_NO_2_ × C_2_H_2_O_4_ (MW = 469.56). ^1^H NMR (500 MHz, DMSO-*d*_6_) δ: 7.70 (d, *J* = 8.59 Hz, 2H), 7.58–7.66 (m, 3H), 7.45–7.56 (m, 2H), 7.04 (d, *J* = 8.88 Hz, 2H), 4.03 (t, *J* = 6.30 Hz, 2H), 3.32 (d, *J* = 10.02 Hz, 2H), 2.86–3.01 (m, 2H), 2.78 (br. s., 2H), 1.67–1.87 (m, 4H), 1.50–1.66 (m, 3H), 1.21–1.47 (m, 6H), 0.78–0.94 (m, 3H). ^13^C NMR (126 MHz, DMSO-*d*_6_) δ: 194.9, 165.4, 163.0, 138.3, 132.7, 132.6, 129.8, 129.7, 129.0, 114.8, 68.3, 56.0, 51.8, 31.1, 31.0, 31.0, 30.9, 28.8, 28.6, 28.5, 26.4, 25.5, 23.7, 21.5, 21.4, 21.4. LC-MS: purity 100% t_R_ = 5.72, (ESI) *m*/*z* [M + H]^+^ 380.35. Anal. calcd. for C_27_H_35_NO_6_: C, 69.06; H, 7.51; N, 2.98%. Found: C, 69.46; H, 7.50; N, 3.08%.

**(2-((5-(Azepan-1-yl)pentyl)oxy)phenyl)(phenyl)methanone hydrogen oxalate (26)**

From (2-(5-bromopentyloxy)phenyl)phenyl)methanone (0.87 g, 2.5 mmol) and azepane (0.50 g, 5 mmol). Yield: 0.49 g (43%). Mp: 143–146 °C. C_24_H_31_NO_2_ × C_2_H_2_O_4_ (MW = 455.53). ^1^H NMR (500 MHz, DMSO-*d*_6_) δ: 7.55–7.67 (m, 3H), 7.42–7.53 (m, 3H), 7.32 (dd, *J*_1_ = 1.15, *J*_2_ = 7.45 Hz, 1H), 7.11 (d, *J* = 8.59 Hz, 1H), 7.05 (t, *J* = 7.16 Hz, 1H), 3.86 (t, *J* = 6.01 Hz, 2H), 3.10 (br. s., 4H), 2.68–2.80 (m, 2H), 1.72 (br. s., 4H), 1.55 (br. s., 4H), 1.36–1.47 (m, 2H), 1.25–1.36 (m, 2H), 0.84 (quin, *J* = 7.59 Hz, 2H). ^13^C NMR (126 MHz, DMSO-*d*_6_) δ: 196.7, 165.4, 156.7, 138.3, 133.6, 132.8, 129.5, 129.5, 129.1, 129.0, 121.1, 113.2, 67.8, 56.6, 53.9, 28.3, 26.6, 23.5, 22.8. LC-MS: purity 100% t_R_ = 5.33, (ESI) *m*/*z* [M + H]^+^ 366.29. Anal. calcd. for C_27_H_35_NO_6_: C, 68.55; H, 7.30; N, 3.07%. Found: C, 68.71; H, 7.51; N, 3.10%.

**(3-((5-(Azepan-1-yl)pentyl)oxy)phenyl)(phenyl)methanone hydrogen oxalate (27)**

From (3-(5-bromopentyloxy)phenyl)phenyl)methanone (0.87 g, 2.5 mmol) and azepane (0.50 g, 5 mmol). Yield: 0.47 g (41%). Mp: 95–98 °C. C_24_H_31_NO_2_ × C_2_H_2_O_4_ (MW = 455.53). ^1^H NMR (300 MHz, DMSO-*d*_6_) δ: 7.62–7.78 (m, 3H), 7.52–7.61 (m, 2H), 7.38–7.50 (m, 1H), 7.16–7.31 (m, 3H), 4.02 (t, *J* = 6.16 Hz, 2H), 3.10–3.35 (m, 4H), 2.93–3.07 (m, 2H), 1.62–1.83 (m, 8H), 1.57 (br. s., 4H), 1.33–1.48 (m, 2H). ^13^C NMR (126 MHz, DMSO-*d*_6_) δ: 196.1, 165.4, 159.1, 138.9, 137.5, 133.3, 130.3, 130.1, 129.1, 122.6, 119.6, 115.2, 67.9, 56.6, 53.9, 28.6, 26.6, 23.8, 23.5, 23.3. LC-MS: purity 99.25% t_R_ = 5.54, (ESI) *m*/*z* [M + H]^+^ 366.35. Anal. calcd. for C_26_H_33_NO_6_: C, 68.55; H, 7.30; N, 3.07%. Found: C, 68.48; H, 6.94; N, 3.10%.

**(4-((5-(Azepan-1-yl)pentyl)oxy)phenyl)(phenyl)methanone hydrogen oxalate (28)**

From (4-(5-bromopentyloxy)phenyl)phenyl)methanone (1.73 g, 5 mmol) and azepane (0.99 g, 10 mmol). Yield: 1.10 g (48%). Mp: 137–140 °C. C_24_H_31_NO_2_ × C_2_H_2_O_4_ (MW = 455.53). ^1^H NMR (500 MHz, DMSO-*d*_6_) δ: 7.70 (d, *J* = 8.88 Hz, 2H), 7.58–7.67 (m, 3H), 7.47–7.56 (m, 2H), 7.05 (d, *J* = 8.88 Hz, 2H), 4.05 (t, *J* = 6.30 Hz, 2H), 3.16 (br. s., 4H), 2.94–3.05 (m, 2H), 1.62–1.82 (m, 8H), 1.56 (br. s., 4H), 1.40 (quin, *J* = 7.52 Hz, 2H). ^13^C NMR (126 MHz, DMSO-*d*_6_) δ: 194.9, 165.4, 162.9, 138.3, 132.7, 132.6, 129.8, 129.0, 114.9, 68.1, 56.6, 53.9, 28.5, 26.6, 23.8, 23.5, 23.3. LC-MS: purity 100% t_R_ = 5.37, (ESI) *m*/*z* [M + H]^+^ 366.37. Anal. calcd. for C_26_H_33_NO_6_: C, 68.55; H, 7.30; N, 3.07%. Found: C, 68.18; H, 7.38; N, 3.03%.

**(4-Chlorophenyl)(4-(5-(azepan-1-yl)pentyloxy)phenyl)methanone hydrogen oxalate (29)**

From (4-(5-bromopentyloxy)phenyl)(4-chlorophenyl)methanone (0.38 g, 1 mmol) and azepane (0.20 g, 2 mmol). Yield: 0.20 g (41%). Mp: 131–132 °C. C_24_H_30_NO_2_Cl × C_2_H_2_O_4_ (MW = 489.99). ^1^H NMR (400 MHz, DMSO-*d*_6_) δ: 7.73 (dd, *J*_1_ = 8.61, *J*_2_ = 13.69 Hz, 4H), 7.56–7.66 (m, 2H), 7.09 (d, *J* = 9.00 Hz, 2H), 4.10 (t, *J* = 6.26 Hz, 2H), 3.20 (br. s., 4H), 2.99–3.10 (m, 2H), 1.66–1.89 (m, 8H), 1.60 (br. s., 4H), 1.37–1.52 (m, 2H). ^13^C NMR (101 MHz, DMSO-*d*_6_) δ: 193.7, 165.2, 163.0, 137.4, 136.9, 132.7, 131.6, 129.4, 129.0, 114.9, 68.1, 56.6, 54.0, 28.4, 26.5, 23.8, 23.5, 23.2. LC-MS: purity 100% t_R_ = 6.08, (ESI) *m*/*z* [M + H]^+^ 400.15. Anal. calcd. for C_26_H_32_NO_6_Cl: C, 63.73; H, 6.58; N, 2.86%. Found: C, 63.94; H, 6.44; N, 2.87%.

**(4-Fluorophenyl) (4-((5-(azepan-1-yl)pentyl)oxy)phenyl)methanone hydrogen oxalate (30)**

From (4-(5-bromopentyloxy)phenyl)(4-fluorophenyl)methanone (0.36 g, 1 mmol) and azepane (0.20 g, 2 mmol). Yield: 0.20 g (42%). Mp: 124–127 °C. C_24_H_30_NO_2_F × C_2_H_2_O_4_ (MW = 473.53). ^1^H NMR (400 MHz, DMSO-*d*_6_) δ: 7.63–7.85 (m, 4H), 7.38 (t, *J* = 8.80 Hz, 2H), 7.09 (d, *J* = 9.00 Hz, 2H), 4.09 (t, *J* = 6.26 Hz, 2H), 3.15–3.29 (m, 4H), 2.92–3.10 (m, 2H), 1.65–1.89 (m, 8H), 1.60 (br. s., 4H), 1.36–1.49 (m, 2H). ^13^C NMR (101 MHz, DMSO-*d*_6_) δ: 193.5, 165.2, 163.5, 162.9, 134.8, 134.7, 132.6, 132.6, 132.5, 129.6, 116.1, 115.9, 114.8, 68.1, 56.6, 53.9, 28.5, 26.6, 23.8, 23.4, 23.2. LC-MS: purity 95.70% t_R_ = 5.51, (ESI) *m*/*z* [M + H]^+^ 384.20. Anal. calcd. for C_26_H_32_NO_6_F: C, 65.94; H, 6.81; N, 2.96%. Found: C, 66.38; H, 7.02; N, 2.88%.

**(2-((6-(Azepan-1-yl)hexyl)oxy)phenyl)(phenyl)methanone hydrogen oxalate (31)**

From (2-(6-bromohexyloxy)phenyl)phenyl)methanone (0.9 g, 2.5 mmol) and azepane (0.5 g, 5 mmol). Yield: 0.26 g (22%). Mp: 121–124 °C. C_25_H_33_NO_2_ × C_2_H_2_O_4_ (MW = 469.56). ^1^H NMR (400 MHz, DMSO-*d*_6_) δ: 7.61–7.72 (m, 3H), 7.47–7.58 (m, 3H), 7.36 (dd, *J*_1_ = 1.56, *J*_2_ = 7.43 Hz, 1H), 7.15 (d, *J* = 8.61 Hz, 1H), 7.09 (t, *J* = 7.43 Hz, 1H), 3.90 (t, *J* = 5.87 Hz, 2H), 3.07–3.26 (m, 4H), 2.80–2.97 (m, 2H), 1.78 (br. s., 4H), 1.60 (br. s., 4H), 1.41–1.54 (m, 2H), 1.29–1.37 (m, 2H), 1.06 (quin, *J* = 7.53 Hz, 2H), 0.82–0.97 (m, 2H). ^13^C NMR (101 MHz, DMSO-*d*_6_) δ: 196.6, 165.2, 156.7, 138.2, 133.5, 132.7, 129.4, 129.0, 121.0, 113.1, 68.0, 56.6, 53.9, 28.6, 26.5, 26.2, 25.1, 23.9, 23.5. LC-MS: purity 100% t_R_ = 5.64, (ESI) *m*/*z* [M + H]^+^ 380.27. Anal. calcd. for C_27_H_35_NO_6_: C, 69.06; H, 7.51; N, 2.98%. Found: C, 68.98; H, 7.52; N, 2.99%.

**(3-((6-(Azepan-1-yl)hexyl)oxy)phenyl)(phenyl)methanone hydrogen oxalate (32)**

From (3-(6-bromohexyloxy)phenyl)phenyl)methanone (1.81 g, 5 mmol) and azepane (0.99 g, 10 mmol). Yield: 0.20 g (44%). Mp: 118–121 °C. C_25_H_33_NO_2_ × C_2_H_2_O_4_ (MW = 469.57). ^1^H NMR (500 MHz, DMSO-*d*_6_) δ: 7.68–7.73 (m, 2H), 7.60–7.67 (m, 1H), 7.48–7.57 (m, 2H), 7.39–7.46 (m, 1H), 7.17–7.24 (m, 3H), 3.98 (t, *J* = 6.44 Hz, 2H), 3.15 (br. s., 4H), 2.89–3.00 (m, 2H), 1.65–1.79 (m, 6H), 1.49–1.64 (m, 6H), 1.40 (quin, *J* = 7.52 Hz, 2H), 1.29 (quin, *J* = 7.37 Hz, 2H). ^13^C NMR (126 MHz, DMSO-*d*_6_) δ: 196.1, 165.3, 159.1, 138.9, 137.5, 133.3, 130.3, 130.1, 129.1, 122.6, 119.6, 115.1, 68.1, 56.7, 54.0, 28.9, 26.6, 26.4, 25.6, 24.1, 23.5. LC-MS: purity 100% t_R_ = 5.89, (ESI) *m*/*z* [M + H]^+^ 380.36. Anal. calcd. for C_27_H_35_NO_6_: C, 69.06; H, 7.51; N, 2.98%. Found: C, 68.57; H, 7.77; N, 2.96%.

**(4-((6-(Azepan-1-yl)hexyl)oxy)phenyl)(phenyl)methanone hydrogen oxalate (33)**

From (4-(6-bromohexyloxy)phenyl)phenyl)methanone (1.81 g, 5 mmol) and azepane (0.99 g, 10 mmol). Yield: 0.94 g (40%). Mp: 128–131 °C. C_25_H_33_NO_2_ × C_2_H_2_O_4_ (MW = 469.56). ^1^H NMR (500 MHz, DMSO-*d*_6_) δ: 7.70 (d, *J* = 8.88 Hz, 2H), 7.58–7.67 (m, 3H), 7.46–7.55 (m, 2H), 7.04 (d, *J* = 8.88 Hz, 2H), 4.04 (t, *J* = 6.30 Hz, 2H), 3.15 (br. s., 4H), 2.91–3.03 (m, 2H), 1.67–1.79 (m, 6H), 1.59–1.67 (m, 2H), 1.55 (br. s., 4H), 1.41 (quin, *J* = 7.45 Hz, 2H), 1.25–1.35 (m, 2H). ^13^C NMR (126 MHz, DMSO-*d*_6_) δ: 194.9, 165.4, 163.0, 138.3, 132.7, 132.6, 129.8, 129.7, 129.0, 114.9, 68.3, 56.7, 53.9, 28.8, 26.6, 26.4, 25.5, 24.0, 23.5. LC-MS: purity 97.70% t_R_ = 5.73, (ESI) *m*/*z* [M + H]^+^ 381.36. Anal. calcd. for C_27_H_35_NO_6_: C, 69.06; H, 7.51; N, 2.98%. Found: C, 68.97; H, 7.77; N, 3.05%.

**(4-Chlorophenyl)(4-((6-(azepan-1-yl)hexyl)oxy)phenyl)methanone hydrogen oxalate (34)**

From (4-(6-bromohexyloxy)phenyl)(4-chlorophenyl)methanone (0.49 g, 1.25 mmol) and azepane (0.25 g, 2.5 mmol). Yield: 0.22 g (35%). Mp: 143–145 °C. C_25_H_32_NO_2_Cl × C_2_H_2_O_4_ (MW = 504.01). ^1^H NMR (400 MHz, DMSO-*d*_6_) δ: 7.72 (dd, *J*_1_ = 8.61, *J*_2_ = 12.13 Hz, 4H), 7.57–7.68 (m, 2H), 7.09 (d, *J* = 8.61 Hz, 2H), 4.08 (t, *J* = 6.26 Hz, 2H), 3.10–3.32 (m, 4H), 2.94–3.08 (m, 2H), 1.55–1.90 (m, 12H), 1.46 (quin, *J* = 7.34 Hz, 2H), 1.28–1.40 (m, 2H). ^13^C NMR (101 MHz, DMSO-*d*_6_) δ: 193.7, 165.2, 163.0, 137.4, 136.9, 132.7, 131.6, 129.4, 129.0, 114.9, 68.3, 56.7, 53.9, 28.7, 26.5, 26.3, 25.5, 24.0, 23.4. LC-MS: purity 100% t_R_ = 6.34, (ESI) *m*/*z* [M + H]^+^ 414.17. Anal. calcd. for C_27_H_34_NO_6_Cl: C, 64.34; H, 6.80; N, 2.78%. Found: C, 64.79; H, 6.80; N, 2.75%.

**(4-((6-(Azepan-1-yl)hexyl)oxy)phenyl)(4-fluorophenyl)methanone hydrogen oxalate (35)**

From (4-(6-bromohexyloxy)phenyl)(4-fluorophenyl)methanone (0.38 g, 1 mmol) and azepane (0.20 g, 2 mmol). Yield: 0.16 g (33%). Mp: 111–114 °C. C_25_H_32_NO_2_F × C_2_H_2_O_4_ (MW = 473.53). ^1^H NMR (400 MHz, DMSO-*d*_6_) δ: 7.69–7.84 (m, 4H), 7.39 (t, *J* = 8.80 Hz, 2H), 7.09 (d, *J* = 9.00 Hz, 2H), 4.09 (t, *J* = 6.26 Hz, 2H), 3.19 (br. s., 4H), 2.92–3.07 (m, 2H), 1.54–1.87 (m, 12H), 1.46 (quin, *J* = 7.24 Hz, 2H), 1.28–1.39 (m, 2H). ^13^C NMR (101 MHz, DMSO-*d*_6_) δ: 193.5, 166.0, 165.1, 163.5, 162.9, 134.8, 134.7, 132.6, 132.5, 129.6, 116.1, 115.9, 114.8, 68.2, 56.7, 54.0, 28.7, 26.5, 26.3, 25.5, 24.0, 23.5. LC-MS: purity 94.57% t_R_ = 5.67, (ESI) *m*/*z* [M + H]^+^ 398.15. Anal. calcd. for C_27_H_34_NO_6_F: C, 66.51; H, 7.03; N, 2.87%. Found: C, 67.04; H, 7.13; N, 2.91%.

### 4.2. Biochemical Assays

#### 4.2.1. H_3_R Affinity

Affinity to *h*H_3_R stably expressed in HEK293 cells was evaluated in a radioligand binding assay as described previously [25]. [^3^H]-*N^α^*-methylhistamine was used as the radioligand and pitolisant (10 µM) was used to define nonspecific binding. The radioactivity was counted in a MicroBeta Trilux counter (PerkinElmer). Data were fitted to a one-site curve-fitting equation with Prism 6 (GraphPad Software, San Diego, CA, USA) and *K_i_* values were calculated from IC_50_ values (from at least three experiments performed in duplicates) according to the Cheng−Prusoff equation [52].

#### 4.2.2. Inhibition of Cholinesterases

The target compounds were tested for their inhibitory potency against cholinesterases using Ellman’s protocol [41], modified for 96-well microplates. Human BuChE from human serum was provided by Vivonics (Bedford, MA, USA). All the other reagents were purchased from Sigma–Aldrich (Steinheim, Germany). The enzymes were prepared as 5 U/mL aqueous stock solutions and diluted before use to a final concentration of 0.384 U/mL. Then 20 μL of prepared enzyme solutions (AChE or BuChE) were added to the reaction mixture in the wells, containing 25 μL of the target compound (or water in case of blank samples), 200 μL of 0.1 M phosphate buffer (pH = 8.0) and 20 μL of 5,5’-dithiobis-(2-nitrobenzoic acid) DTNB (0.0025M). All those reagents were preincubated for 5 min at 25 °C for animal enzymes or 36 °C for human ones. The enzymatic reaction was initiated by the addition of 20 μL of substrate acetylthiocholine iodide ATC (0.00375M) or butyrylthiocholine iodide BTC (0.00375M) solutions (depending on the enzyme used). After 5 min of incubation, changes in absorbance were measured at 412 nm, using EnSpire multimode microplate reader (PerkinElmer, Waltham, MA, USA). Target compounds were tested at a screening concentration of 10 μM. Percent of enzyme inhibition was calculated based on the formula 100 − (S/B) × 100, where S and B were the respective enzyme activities with and without the test compound, respectively. For the most potent compounds, with at least 50% of the enzyme inhibitory activity, IC_50_ values were determined. Calculations were based on the absorbance measured at six different concentrations of the inhibitor, then converted to the % of enzyme inhibition using the above-presented formula. The obtained percentages of enzyme inhibition were plotted against the applied inhibitor concentrations, using nonlinear regression (GraphPad Prism 9; GraphPad Software, San Diego, CA, USA). Tacrine was tested as a reference compound. All the experiments were performed in triplicate.

#### 4.2.3. Kinetics of ChEs Inhibition

The kinetic studies were performed with compound **6**, using Ellman’s method [41], modified for 96-well microplates [26]. The inhibitor was tested in the concentrations giving enzyme activities between 30% and 80%. For each concentration of the inhibitor, substrate (ATC/BTC) was added at concentrations of 0.3, 0.24, 0.18, 0.12, 0.06, and 0.04 mM in the wells. The stock solutions of ATC and BTC (0.5 mM) were prepared in water and diluted before use. Each experiment was performed in triplicate. V_max_ and K_m_ values of the Michaelis−Menten kinetics were calculated by nonlinear regression from substrate−velocity curves. Lineweaver–Burk and Cornish–Bowden plots were calculated using linear regression in GraphPad Prism 5 (GraphPad Software, San Diego, CA, USA).

#### 4.2.4. Inhibition of Human MAO B

The precise method was described in [32]. Briefly, tested compounds were screened for *h*MAO B inhibitory activity at 1 μM concentration by the fluorometric method. Para-tyramine (200 μM) was used as a substrate for the enzyme while safinamide (1 μM) and rasagiline (1 μM) were used as reference inhibitors (reversible and irreversible, respectively). Each experiment was performed twice in duplicate.

### 4.3. Docking Studies

#### 4.3.1. Molecular Modeling Studies to Histamine H_3_ Receptor

For docking purposes, MOE v 2019.1 [53] was used. Ligands were built in their ionized forms (protonated N4 piperazine nitrogen, structure charge +1). Bioactive conformations were generated using the OMEGA package [54] (maximum number of conformers = 100; RMS = 0.5). For all compounds, the five lowest energy conformers were selected for docking studies. Possible binding pocket adaptation in presence of certain ligands was examined using the induced fit refinement protocol with **6**. To validate the methods used, the ligand was then redocked with high confidence. The site was then centered on the ligand. Docking to the rigid form of the receptor was performed using standard docking protocol. Ligands were rated according to their position in the binding pocket, the docking score value and interactions with binding pocket amino acids. Ligand–receptor visualizations were generated using Schrödinger Maestro free academic. Dynamics simulations (in time of 600 ps, T = 300 K) were performed using the Nosé-Poincaré-Andersen equations of motion, forcefield AMBER10:EHT; R-Field 1:80, Cutoff (8,10) and performed in MOE v 2019.1 [53].

#### 4.3.2. Molecular Modeling Studies to AChE, BuChE and MAO B

The tested compounds were drawn in Marvin and saved in smiles format. Subsequently, they were prepared with a LigPrep module in pH 7.4 ± 0.2 with an ionizer function, and available isomers were generated. Docking studies were carried out according to the earlier validated procedures [44]. Ligands were docked into the: acetylcholinesterase (PDB code: 1EVE), butyrylcholinesterase (PDB code: 1P0I) and human monoamine oxidase B (PDB code: 4A79). The crystal structures of proteins were prepared with Hermes: the protonation of histidines was set at Nε atoms, and hydrogens were added. Ligand and water molecules were removed from the complexes, except for water molecules with numbers: 1159, 1249 and 1254 in AChE. Binding sites for docking were marked within 10 Å, 20 Å and 12 Å from the reference compounds: donepezil–AChE, butyrate–BuChE and pioglitazone for MAO B. For calculations, standard parameters of the genetic algorithm were used. The population size was defined as 100 and the operation number was 100,000. For each compound 10 poses were obtained. Results were analyzed in PyMOL software. For molecular dynamics simulation, all-atom systems and input files were prepared with the CHARMM-GUI online server. The complexes of protein with ligands were prepared as follows: solvation was performed with TIP3P water molecules, and Na^+^ and Cl^−^ ions were added (0.15 M NaCl). For system equilibration, the one-step protocol implemented in CHARMM-GUI was used. Molecular dynamics simulations were performed with NAMD version 2.13 at a temperature of 303.15 K. MD timesteps were selected as 2fs, and the total time was set as 10 ns. The calculation was performed with a CHARMM36m force field. Results analysis was performed with the VMD program.

### 4.4. ADMET Properties

#### 4.4.1. Metabolic Stability

The in silico prediction of the most possible metabolic pathways was conducted by MetaSite 6.0.1. Software (Molecular Discovery Ltd., Borehamwood, UK). The in vitro metabolic stability determination of compound **6** was performed with mouse liver microsomes (MLMs, Sigma-Aldrich, St. Louis, MO, USA). The compound was incubated for 120 min at 37 °C in TRIS-HCl buffer (pH 7.4) with the addition of NADPH Regeneration System (Promega, Madison, WI, USA). The LC/MS analysis and MS/MS fragmentations were used as a tool for the determination of the substrate remaining and the structures of the metabolites.

#### 4.4.2. Neuroprotection

Neuroprotection studies were performed according to the procedures described previously [46]. In brief, SH-SY5Y CRL-2266 ™ (American Type Culture Collection, Manassas, VG, USA) were seeded in a microplate at a concentration of 2.5 × 10^4^ cells/well in 100 μL culture medium and cultured for 24 h at 37 °C and 5% CO_2_. The cells were preincubated first for 1h with compound **6** (10 µM) or with the reference neuroprotectant salsolinol (SAL). Next, H_2_O_2_ was added at a final concentration of 300 μM and the cells were placed into the incubator. After 24 h of the compound co-incubation with H_2_O_2_, the CellTiter 96^®^ AQueous Non-Radioactive Cell Proliferation Assay (MTS) and CytoTox-ONE™ Homogeneous Membrane Integrity Assay (second independent experiment) were performed. Both tests were purchased from Promega (Madison, WI, USA). The absorbance and fluorescence were measured using a microplate reader EnSpire (PerkinElmer, Waltham, MA USA) at 490 nm. All results were shown as mean ± SD. The statistical significances were calculated by GraphPad Prism 6.0 software.

#### 4.4.3. Permeability

The passive transport of compound **6** through the cell membranes was estimated by Pre-coated PAMPA Plate System Gentest ™ provided by Corning (Tewksbury, MA, USA) and was carried out according to the procedure previously described [32]. The assay was carried out according to the manufacturer’s instruction and the permeability coefficient Pe was counted on the basis of the provided equations. The concentrations of a tested compound in the apical and basolateral wells were determined using the LC/MS analysis with an internal standard. The results were compared to the high permeable reference caffeine (CFN).

#### 4.4.4. Hepatotoxicity

Hepatoma HepG2 HB-8065 ™ (American Type Culture Collection, Manassas, VG, USA) cells were seeded in a microplate at a concentration of 1.0 × 10^4^ cells/well in 100 μL and incubated for 24 h. Compound **6** was added next to the cells in the six concentrations: 0.1, 1, 10, 25, 50 and 100 µM and incubated for 72 h. The cell viability was defined by an MTS assay provided by Promega (Madison, WI, USA). The results were compared to the reference toxin DX which was implemented at 1 µM. A multifunctional reader EnSpire (PerkinElmer, Waltham, MA, USA) was used to measure the absorbance.

### 4.5. In Vivo Experiments

All experimental procedures were performed according to the European Union Directive of 22 September 2010 (2010/63/EU) and Polish legislation concerning animal care and use and were approved by the Local Ethics Committee for Experiments on Animals in Kraków, Poland (Resolution No. 179/2017 and 279/2019).

#### 4.5.1. Passive Avoidance Test

The experimental procedure was performed based on the previously described study [30]. The main equipment used in the test was the step-through passive avoidance task apparatus (LE872, Panlab Harvard Apparatus, Spain) containing the dark and the light compartment. In the acquisition session on the first day of the test, each mouse was placed in the light compartment and punished by an electric foot shock (0.2 mA for 2 s, Shocker LE100-26) when it entered into the dark compartment. In the retention session (24 h later) each animal was observed up to 180 s and the latency to the entrance to the dark compartment was measured. The latency time to the entrance to the dark compartment in the retention session close to the maximum possible value was considered proper memory performance.

#### 4.5.2. Formalin Test in Mice

The mice were administered (*ip*) the tested compound or vehicle, and after 30 min, 20 μL of a 2.5% formalin solution was injected intraplantarly into the right hind paw. Immediately after the formalin injection, the animals were placed individually into glass beakers and were observed over the next 30 min. The time that the animal spent licking or biting the injected paw was measured during selected intervals: 0–5 min (I phase), and 15–30 min (II phase) post-formalin injection [55,56].

## Figures and Tables

**Table 1:** Structures and in vitro activity of tested compounds (**2**–**35**).

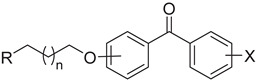								
R	Compd	n	Position ^a^	X	*h*H_3_R *K_i_* [nM] ^b^	*ee*AChE IC_50_ [μM] ^c^ (% Inh. at 10 µM)	*eq*BuChE IC_50_ [μM] ^d^ (% Inh. at 10 µM)	*h*MAO B (%Inh. at 1 µM) ^e^
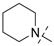	**2**	3	*ortho*	H	371 [120;1147]	22% ± 1	1.607 ± 0.060	23% ± 2
	**3**	3	*meta*	H	210 [101;435]	7.754 ± 0.329	4.006 ± 0.067	15% ± 1
	**4**	3	*para*	H	36 * [10;30]	41% ± 1	2.477 ± 0.040	44% ± 1
	**5**	3	*para*	4-Cl	20 [3;141]	2.870 ± 0.071	1.685 ± 0.017	18% ± 0
	**6**	3	*para*	4-F	8 [1;62]	2.306 ± 0.004 *h*AChE ^f^ 9.585 ± 0.220	0.172 ± 0.005 *h*BuChE ^g^ 1.155 ± 0.034	25% ± 4
	**7**	4	*ortho*	H	161 [92;280]	3% ± 0	2.108 ± 0.032	12% ± 0
	**8**	4	*meta*	H	76 * [54;108]	3.627 ± 0.111	2.096 ± 0.060	15% ± 3
	**9**	4	*para*	H	41 * [26;66]	3.733 ± 0.085	1.781 ± 0.034	9% ± 2
	**10**	4	*para*	4-Cl	25 [11;58]	2.669 ± 0.094	2.457 ± 0.061	39% ± 2
	**11**	4	*para*	4-F	26 [9;72]	3.098 ± 0.009	0.419 ± 0.002	28% ± 3
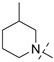	**12**	3	*ortho*	H	208 [80;543]	16% ± 2	1.607 ± 0.061	9% ± 1
	**13**	3	*meta*	H	78 [39;130]	3.790 ± 0.104	3.424 ± 0.078	8% ± 6
	**14**	3	*para*	H	40 * [13;119]	3.180 ± 0.083	3.781 ± 0.061	9% ± 4
	**15**	3	*para*	4-Cl	37 [22;60]	2.646 ± 0.087	2.562 ± 0.050	19% ± 9
	**16**	3	*para*	4-F	12 [2;71]	2.023 ± 0.006	0.311 ± 0.006	20% ± 3
	**17**	4	*ortho*	H	152 [72;322]	20% ± 2	2.498 ± 0.028	8% ± 1
	**18**	4	*meta*	H	110 * [62;196]	2.002 ± 0.030	2.895 ± 0.077	27% ± 7
	**19**	4	*para*	H	53 * [31;89]	2.106 ± 0.050	1.978 ± 0.034	7% ± 1
	**20**	4	*para*	4-Cl	44 [26;75]	2.737 ± 0.098	2.523 ± 0.031	17% ± 2
	**21**	4	*para*	4-F	75 [36;154]	1.999 ± 0.046	2.560 ± 0.040	23% ± 6
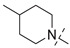	**22**	3	*meta*	H	298 [192;462]	4.451 ± 0.094	1.705 ± 0.046	32% ± 2
	**23**	3	*para*	H	67 [61;74]	2.603 ± 0.072	1.567 ± 0.035	27% ± 2
	**24**	4	*meta*	H	106 [89;127]	2.457 ± 0.070	1.063 ± 0.043	20% ± 3
	**25**	4	*para*	H	53 [34;82]	2.692 ± 0.047	1.556 ± 0.050	22% ± 12
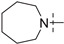	**26**	3	*ortho*	H	302 [46;1995]	24% ± 3	0.384 ± 0.008	3% ± 0
	**27**	3	*meta*	H	307 [188;502]	3.661 ± 0.092	0.901 ± 0.010	14% ± 2
	**28**	3	*para*	H	39 * [11;142]	4.630 ± 0.100	0.678 ± 0.007	29% ± 1
	**29**	3	*para*	4-Cl	66 [5;892]	2.244 ± 0.035	0.676 ± 0.003	26% ± 1
	**30**	3	*para*	4-F	13 [2;92]	1.110 ± 0.003	0.224 ± 0.003	30% ± 1
	**31**	4	*ortho*	H	162 [82;319]	11% ± 1	0.317 ± 0.003	5% ± 1
	**32**	4	*meta*	H	70 * [44;170]	1.551 ± 0.034	0.616 ± 0.002	17% ± 4
	**33**	4	*para*	H	41 * [33;50]	1.810 ± 0.038	1.281 ± 0.038	23% ± 1
	**34**	4	*para*	4-Cl	133 [35;508]	2.033 ± 0.018	0.867 ± 0.002	30% ± 2
	**35**	4	*para*	4-F	35 [6;204]	1.654 ± 0.004	0.161 ± 0.004	24% ± 0
Reference	**pitolisant**				12 ± 3 **	3% ± 0	8.400 ± 0.180	2% ± 3
	**tacrine**				nt	0.024 ± 0.001	0.015 ± 0.001	nt
	**rasagiline ^h^**				nt	nt	nt	25 ± 7
	**safinamide**				nt	nt	nt	98% ± 4 7.6 ± 1 ^i^

^a^ of the alkoxyl chain, ^b^ [^3^H]*N^α^*-Methylhistamine binding assay in HEK293 cells stably expressing the human H_3_R; mean value within the 95% confidence interval (CI) for three repetitions, ^c^ IC_50_ inhibitory concentration of *electric eel* AChE; mean value ± standard error of the mean (SEM) of triplicate independent experiments, ^d^ IC_50_ inhibitory concentration of BuChE from *equine serum*; mean value ± standard error of the mean (SEM) of triplicate independent experiments, ^e^ fluorometric MAO assay; mean value ± standard error of the mean (SEM) of two independent experiments, ^f^ *human recombinant* AChE, ^g^ BuChE from *human serum*, ^h^ IC_50_ value [nM] ± standard error of the mean (SEM) of four independent experiments in duplicates, ^i^ IC_50_ value [nM] ± standard error of the mean (SEM) of five independent experiments in duplicates; data from [32]; * data from [31]; ** data from [33], nt, not tested.

**Table 2:** Metabolic stability summary: the molecular masses and metabolic pathways of **6** after incubation with mouse liver microsomes (MLMs). Main metabolic pathways are marked in red.

Substrate	Molecular Mass (*m*/*z*)	% Remaining	Molecular Mass of the Metabolite (*m*/*z*)	Metabolic Pathway
**6**	370.33	65.95	372.39 (M1) 386.35 (M2) 388.41 (M3)	reduction of carbonyl group hydroxylation reduction of carbonyl group, hydroxylation
**Verapamil** (control) *	455.54	23.93	441.42 (M1)	demethylation
			441.42 (M2)	demethylation
			291.35 (M3)	defragmentation
			293.34 (M4)	defragmentation/hydroxylation
			277.33 (M5)	defragmentation

* data from ref. [47] obtained according to the same procedure as used for **6**.

**Table 3:** Permeability coefficient of compound **6**.

Compound	*P_e_* ^n,o^ [10^−6^ cm/s] ± SD
**6**	6.3 ± 0.6
Caffeine	15.1 ± 0.4

^n^ tested in triplicate; ^o^ for permeable compounds *P_e_* is equal or higher than 1.5 × 10^−6^ cm/s [49].

## Data Availability

Data are available from the authors upon request.

## Supplementary Materials

The following supporting information can be downloaded at: https://www.mdpi.com/article/10.3390/molecules28010238/s1, Figure S1: RMSD changes for ligand **6** within AChE active site during molecular dynamics simulation; Figure S2: Distances between ligand **6** (ether O atom) and Tyr121 (OH group) from AChE during molecular dynamics simulation; Figure S3: RMSD changes for ligand **6** within BuChE active site during molecular dynamics simulation; Figure S4: Changes of ligand **6** positions within MAO B active site during molecular dynamics simulation; Figure S5: Distances between ligand **6** (CO group) and selected residues: Gln206(NH), Tyr398(OH), Tyr435(OH) from MAO B during molecular dynamics simulation; Figure S6: MS/MS ion fragmentation analyses of **6** and its metabolites.

## Sample Availability

Samples of the compounds are available from the authors.

## Abbreviations

Aβ—amyloid beta, ACh—acetylcholine, AChE—acetylcholinesterase, AD—Alzheimer’s disease, BBB—blood-brain barrier, BuChE—butyrylcholinesterase, CNS—central nervous system, DDI—drug/drug interactions, DX—doxorubicin, *ee*AChE—acetylcholinesterase from *electric eel*, *eq*BuChE—butyrylcholinesterase from *equine serum*, H_3_R—histamine H_3_ receptor, *h*H_3_R—human histamine H_3_ receptor, *h*AChE—human acetylcholinesterase, *h*BuChE—human butyrylcholinesterase, *h*MAO B—human monoamine oxidase B, KE—ketoconazole, MAO B—monoamine oxidase B, MLMs—mouse liver microsomes, MTDL—multi-target-directed ligand, PAMPA—parallel artificial membrane permeability assay, PAS—peripheral anionic site

## References

[1] Handra C., Andreia Coman O., Coman L., Enache T., Stoleru S., Sorescu A.-M., Ghiță I., Fulga I. (2019). The connection between different neurotransmitters involved in cognitive processes. Farmacia.

[2] Cummings J., Lee G., Zhong K., Fonseca J., Taghva K. (2021). Alzheimer’s disease drug development pipeline: 2021. Alzheimer’s Dement. Transl. Res. Clin. Interv..

[3] Alvarez E.O. (2009). The role of histamine on cognition. Behav. Brain Res..

[4] Davies P., Maloney A.J.F. (1976). Selective loss of central cholinergic neurons in Alzheimer’s Disease. Lancet.

[5] Pohanka M. (2011). Cholinesterases, a target of pharmacology and toxicology. Biomed. Pap..

[6] Singh M., Jadhav H.R. (2012). Histamine H3 Receptor Function and Ligands: Recent Developments. Mini Rev. Med. Chem..

[7] Khanfar M.A., Affini A., Lutsenko K., Nikolic K. (2016). Multiple Targeting Approaches on Histamine H3 Receptor Antagonists. Front. Neurosci..

[8] Sadek B., Saad A., Sadeq A., Jalal F., Stark H. (2016). Histamine H3 receptor as a potential target for cognitive symptoms in neuropsychiatric diseases. Behav. Brain Res..

[9] Giacobini E. (2004). Cholinesterase inhibitors: New roles and therapeutic alternatives. Pharmacol. Res..

[10] Brioni J.D., Esbenshade T.A., Garrison T.R., Bitner S.R., Cowart M.D. (2011). Discovery of histamine H3 antagonists for the treatment of cognitive disorders and Alzheimer’s disease. J. Pharmacol. Exp. Ther..

[11] Zlomuzica A., Dere D., Binder S., De Souza Silva M.A., Huston J.P., Dere E. (2016). Neuronal histamine and cognitive symptoms in Alzheimer’s disease. Neuropharmacology.

[12] Bautista-Aguilera O.M., Budni J., Mina F., Medeiros E.B., Deuther-Conrad W., Entrena J.M., Moraleda I., Iriepa I., Marco-Contelles J. (2018). Contilisant, a Tetratarget Small Molecule for Alzheimer’s Disease Therapy Combining Cholinesterase, Monoamine Oxidase Inhibition, and H3R Antagonism with S1R Agonism Profile. J. Med. Chem..

[13] Lopes F.B., Aranha C.M.S.Q., Fernandes J.P.S. (2021). Histamine H3 receptor and cholinesterases as synergistic targets for cognitive decline: Strategies to the rational design of multitarget ligands. Chem. Biol. Drug Des..

[14] Huang W., Tang L., Shi Y., Huang S., Xu L., Sheng R., Wu P., Li J. (2011). Bioorganic & Medicinal Chemistry Searching for the Multi-Target-Directed Ligands against Alzheimer’s disease: Discovery of quinoxaline-based hybrid compounds with AChE, H3R and BACE 1 inhibitory activities. Bioorg. Med. Chem..

[15] Galimberti D., Scarpini E. (2016). Old and new acetylcholinesterase inhibitors for Alzheimer’s disease. Expert Opin. Investig. Drugs.

[16] Pepeu G., Giovannini M.G. (2010). Cholinesterase inhibitors and memory. Chem. Biol. Interact..

[17] Blanco-Silvente L., Castells X., Saez M., Barceló M.A., Garre-Olmo J., Vilalta-Franch J., Capellà D. (2017). Discontinuation, Efficacy, and Safety of Cholinesterase Inhibitors for Alzheimer’s Disease: A Meta-Analysis and Meta-Regression of 43 Randomized Clinical Trials Enrolling 16,106 Patients. Int. J. Neuropsychopharmacol..

[18] Mehta M., Adem A., Sabbagh M. (2012). New acetylcholinesterase inhibitors for alzheimer’s disease. Int. J. Alzheimers Dis..

[19] Łazewska D., Kieć-Kononowicz K. (2014). New developments around histamine H3 receptor antagonists/inverse agonists: A patent review (2010-present). Expert Opin. Ther. Pat..

[20] Maramai S., Benchekroun M., Gabr M.T., Yahiaoui S. (2020). Multitarget Therapeutic Strategies for Alzheimer’s Disease: Review on Emerging Target Combinations. BioMed Res. Int..

[21] Bolognesi M.L., Simoni E., Rosini M., Minarini A., Tumiatti V., Melchiorre C. (2011). Multitarget-Directed Ligands: Innovative Chemical Probes and Therapeutic Tools Against Alzheimer’s Disease. Curr. Top. Med. Chem..

[22] Ramalakshmi N., Remya R.S., Nalini C.N. (2021). Multitarget Directed Ligand Approaches for Alzheimer’s Disease: A Comprehensive Review. Mini Rev. Med. Chem..

[23] Bembenek S.D., Keith J.M., Letavic M.A., Apodaca R., Barbier A.J., Dvorak L., Aluisio L., Miller K.L., Lovenberg T.W., Carruthers N.I. (2008). Lead identification of acetylcholinesterase inhibitors–histamine H3 receptor antagonists from molecular modeling. Bioorg. Med. Chem..

[24] Ghamari N., Dastmalchi S., Zarei O., Arias-Montaño J., Reiner D., Ustun-Alkan F., Stark H., Hamzeh-Mivehroud M. (2020). In silico and in vitro studies of two non-imidazole multiple targeting agents at histamine H3 receptors and cholinesterase enzymes. Chem. Biol. Drug Des..

[25] Incerti M., Flammini L., Saccani F., Morini G., Comini M., Coruzzi M., Barocelli E., Ballabeni V., Bertoni S. (2010). Dual-Acting Drugs: An in vitro Study of Nonimidazole Histamine H3 Receptor Antagonists Combining Anticholinesterase Activity. ChemMedChem.

[26] Bajda M., Łażewska D., Godyń J., Zaręba P., Kuder K., Hagenow S., Łątka K., Stawarska E., Stark H., Kieć-Kononowicz K. (2020). Search for new multi-target compounds against Alzheimer’s disease among histamine H3 receptor ligands. Eur. J. Med. Chem..

[27] Łażewska D., Jończyk J., Bajda M., Szałaj N., Więckowska A., Panek D., Moore C., Kuder K., Malawska B., Kieć-Kononowicz K. (2016). Cholinesterase inhibitory activity of chlorophenoxy derivatives—Histamine H3 receptor ligands. Bioorg. Med. Chem. Lett..

[28] Bajda M., Kuder K.J., Łewska D., Kieć-Kononowicz K., Wiȩckowska A., Ignasik M., Guzior N., Jończyk J., Malawska B. (2012). Dual-Acting Diether Derivatives of Piperidine and Homopiperidine with Histamine H3 Receptor Antagonistic and Anticholinesterase Activity. Arch. Pharm..

[29] Godyń J., Zaręba P., Łażewska D., Stary D., Reiner-Link D., Frank A., Latacz G., Mogilski S., Kaleta M., Doroz-Płonka A. (2021). Cyanobiphenyls: Novel H3 receptor ligands with cholinesterase and MAO B inhibitory activity as multitarget compounds for potential treatment of Alzheimer’s disease. Bioorg. Chem..

[30] Łażewska D., Zaręba P., Godyń J., Doroz-Płonka A., Frank A., Reiner-Link D., Bajda M., Stary D., Mogilski S., Olejarz-Maciej A. (2021). Biphenylalkoxyamine Derivatives–Histamine H3 Receptor Ligands with Butyrylcholinesterase Inhibitory Activity. Molecules.

[31] Alachkar A., Łażewska D., Latacz G., Frank A., Siwek A., Lubelska A., Honkisz-Orzechowska E., Handzlik J., Stark H., Kieć-Kononowicz K. (2018). Studies on Anticonvulsant Effects of Novel Histamine H3R Antagonists in Electrically and Chemically Induced Seizures in Rats. Int. J. Mol. Sci..

[32] Łażewska D., Bajda M., Kaleta M., Zaręba P., Doroz-Płonka A., Siwek A., Alachkar A., Mogilski S., Saad A., Kuder K. (2020). Rational design of new multitarget histamine H3 receptor ligands as potential candidates for treatment of Alzheimer’s Disease. Eur. J. Med. Chem..

[33] Sander K., von Coburg Y., Camelin J.C., Ligneau X., Rau O., Schubert-Zsilavecz M., Schwartz J.C., Stark H. (2010). Acidic elements in histamine H3 receptor antagonists. Bioorg. Med. Chem. Lett..

[34] Wingen K., Stark H. (2013). Scaffold variations in amine warhead of histamine H3 receptor antagonists. Drug Discov. Today Technol..

[35] Miles J.A., Ross B.P. (2020). Recent Advances in Virtual Screening for Cholinesterase Inhibitors. Chem. Neurosci..

[36] Cruz M.I., Cidade H., Pinto M. (2017). Dual/multitargeted xanthone derivatives for Alzheimer’s disease: Where do we stand?. Futur. Med. Chem..

[37] Rizzo S., Cavalli A., Ceccarini L., Bartolini M., Belluti F., Bisi A., Andrisano V., Recanatini M., Rampa A. (2009). Structure–Activity Relationships and Binding Mode in the Human Acetylcholinesterase Active Site of Pseudo-Irreversible Inhibitors Related to Xanthostigmine. ChemMedChem.

[38] Menéndez C.A., Biscussi B., Accordino S., Paula Murray A., Gerbino D.C., Appignanesi G.A. (2017). Design, synthesis and biological evaluation of 1,3-dihydroxyxanthone derivatives: Effective agents against acetylcholinesterase. Bioorg. Chem..

[39] Kou X., Song L., Wang Y., Yu Q., Ju H., Yang A., Shen R. (2020). Design, synthesis and anti-Alzheimer’s disease activity study of xanthone derivatives based on multi-target strategy. Bioorgan. Med. Chem. Lett..

[40] Belluti F., Piazzi L., Bisi A., Gobbi S., Bartolini M., Cavalli A., Valenti P., Rampa A. (2009). Design, synthesis, and evaluation of benzophenone derivatives as novel acetylcholinesterase inhibitors. Eur. J. Med. Chem..

[41] Ellman G.L., Courtney K.D., Andres V., Featherstone R.M. (1961). A new and rapid colorimetric determination of acetylcholinesterase activity. Biochem. Pharmacol..

[42] Szczepańska K., Karcz T., Mogilski S., Siwek A., Kuder K.J., Latacz G., Kubacka M., Hagenow S., Lubelska A., Olejarz A. (2018). Synthesis and biological activity of novel tert-butyl and tert-pentylphenoxyalkyl piperazine derivatives as histamine H3R ligands. Eur. J. Med. Chem..

[43] Levoin N., Calmels T., Poupardin-Olivier O., Labeeuw O., Danvy D., Robert P., Berrebi-Bertrand I., Ganellin C.R., Schunack W., Stark H. (2008). Refined Docking as a Valuable Tool for Lead Optimization: Application to Histamine H3 Receptor Antagonists. Arch. Pharm..

[44] Bajda M., Wiȩckowska A., Hebda M., Guzior N., Sotriffer C.A., Malawska B. (2013). Structure-based search for new inhibitors of cholinesterases. Int. J. Mol. Sci..

[45] Kryger G., Silman I., Sussman J.L. (1999). Structure of acetylcholinesterase complexed with E2020 (Ariceptρ): Implications for the design of new anti-Alzheimer drugs. Structure.

[46] MetaSite.

[47] Wichur T., Godyń J., Góral I., Latacz G., Bucki A., Siwek A., Głuch-Lutwin M., Mordyl B., Śniecikowska J., Walczak M. (2021). Development and crystallography-aided SAR studies of multifunctional BuChE inhibitors and 5-HT6R antagonists with β-amyloid anti-aggregation properties. Eur. J. Med. Chem..

[48] Kurnik-Łucka M., Latacz G., Martyniak A., Bugajski A., Kieć-Kononowicz K., Gil K. (2020). Salsolinol—Neurotoxic or Neuroprotective?. Neurotox. Res..

[49] Chen X., Murawski A., Patel K., Crespi C.L., Balimane P.V. (2008). A novel design of artificial membrane for improving the PAMPA model. Pharm. Res..

[50] Paniagua A.C., Amariles P., Malangu N. (2017). Hepatotoxicity by Drugs. Pharmacokinetics and Adverse Effects of Drugs—Mechanisms and Risks Factors.

[51] Schindler U. (1989). Pre-clinical evaluation of cognition enhancing drugs, Prog. Neuropsychopharmacol. Biol. Psychiatry.

[52] Cheng Y.-C., Prusoff W.H. (1973). Relationship between the inhibition constant (K1) and the concentration of inhibitor which causes 50 per cent inhibition (I50) of an enzymatic reaction. Biochem. Pharmacol..

[53] (2019). Molecular Operating Environment (MOE) 2019.1.

[54] Hawkins P.C.D., Skillman A.G., Warren G.L., Ellingson B.A., Stahl M.T. (2010). Conformer Generation with OMEGA: Algorithm and Validation Using High Quality Structures from the Protein Databank and Cambridge Structural Database. J. Chem. Inf. Model..

[55] Salinas-Abarca A.B., Avila-Rojas S.H., Barragán-Iglesias P. (2017). Formalin injection produces long-lasting hypersensitivity with characteristics of neuropathic pain. Eur. J. Pharmacol..

[56] Muley M.M., Krustev E., Mcdougall J.J., Pain C. (2016). Preclinical Assessment of Inflammatory Pain Models of Cutaneous Pain. CNS Neurosci. Ther..

