# Supplementary material for: Benzophenone Derivatives with Histamine H3 Receptor Affinity and Cholinesterase Inhibitory Potency as Multitarget-Directed Ligands for Possible Therapy of Alzheimer’s Disease
Source: Molecules. 2022 Dec 28;28(1):238. doi: 10.3390/molecules28010238 (PMC9822066; doi:10.3390/molecules28010238)
Supplement: Supplementary file 1 [file molecules-28-00238-s001.zip › molecules-2085369-supplementary.pdf]

Supplementary Materials

Benzophenone Derivatives with Histamine H3 Receptor Affinity and Cholinesterase Inhibitory Potency as Multitarget-Directed Ligands for Possible Therapy of Alzheimer's Disease

Justyna Godyń [1], Paula Zaręba [1], Dorota Stary [1,2], Maria Kaleta [3], Kamil J. Kuder [3], Gniewomir Latacz [3], Szczepan Mogilski [4], David Reiner-Link [5], Annika Frank [5], Agata Doroz-Płonka [3], Agnieszka Olejarz-Maciej [3], Sylwia Sudoł-Tałaj [2,3], Tobias Nolte [3], Jadwiga Handzlik [3], Holger Stark [5], Anna Więckowska [1], Barbara Malawska [1], Katarzyna Kieć-Kononowicz [3], Dorota Łażewska [3,†] and Marek Bajda [1,*,†]

[1] Department of Physicochemical Drug Analysis, Jagiellonian University Medical College, Medyczna 9 St., 30-688 Krakow, Poland
[2] Doctoral School of Medical and Health Sciences, Jagiellonian University Medical College, św. Łazarza 16 St., 31-530 Krakow, Poland
[3] Department of Technology and Biotechnology of Drugs, Jagiellonian University Medical College, Medyczna 9 St., 30-688 Krakow, Poland
[4] Department of Pharmacodynamics, Jagiellonian University Medical College, Medyczna 9 St., 30-688 Krakow, Poland
[5] Institute of Pharmaceutical and Medicinal Chemistry, Heinrich Heine University Duesseldorf, Universitaetsstr. 1, 40225 Duesseldorf, Germany
* Correspondence: marek.bajda@uj.edu.pl
† These authors contributed equally to this work.

1. *In silico* studies

1.1 Analysis of a binding mode within AChE

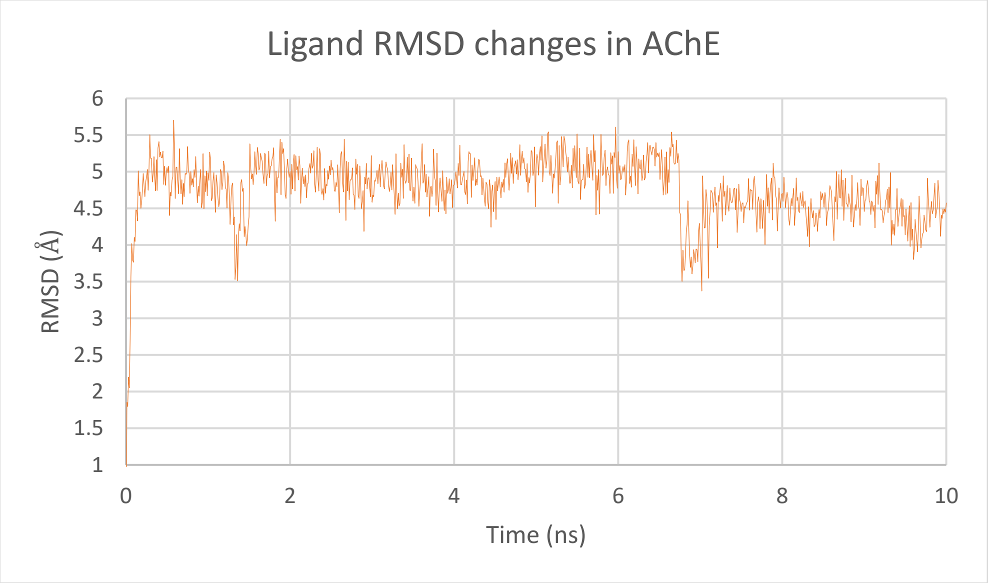

Figure S1. RMSD changes for ligand **6** within AChE active site during molecular dynamics simulation

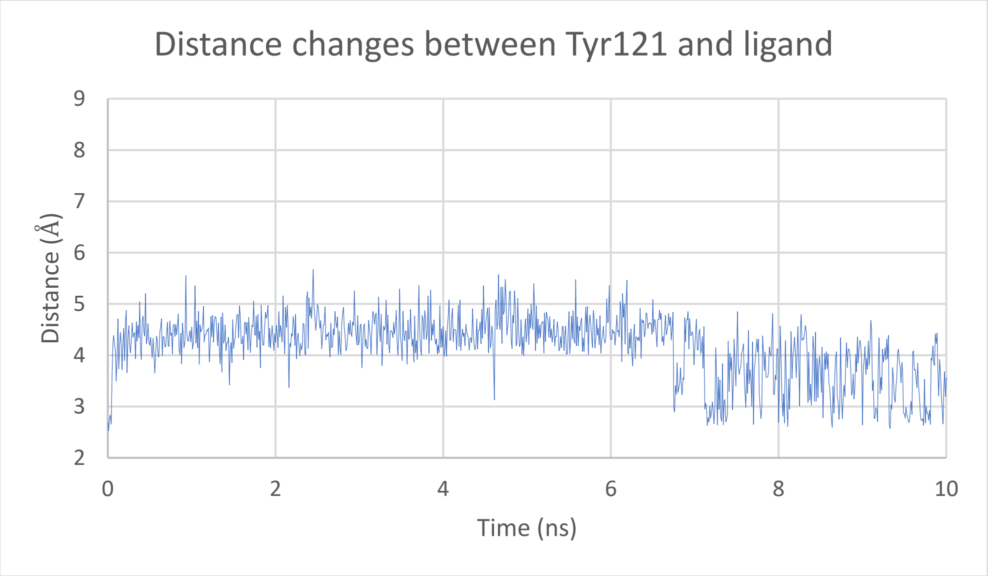

Figure S2. Distances between ligand **6** (ether O atom) and Tyr121 (OH group) from AChE during molecular dynamics simulation

1.2 Analysis of binding mode within BuChE

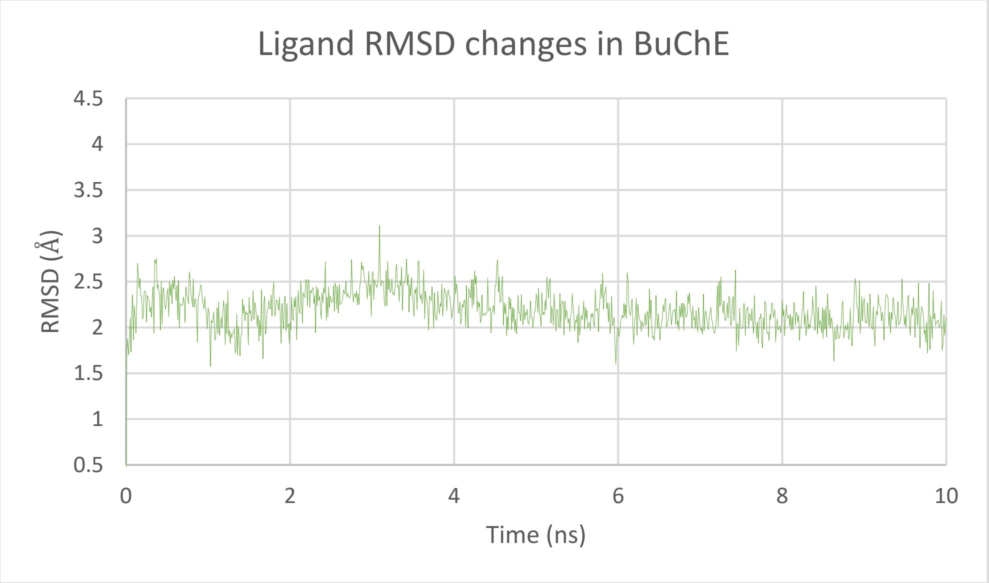

Figure S3. RMSD changes for ligand **6** within BuChE active site during molecular dynamics simulation

1.3 Analysis of binding mode with monoamine oxidase B

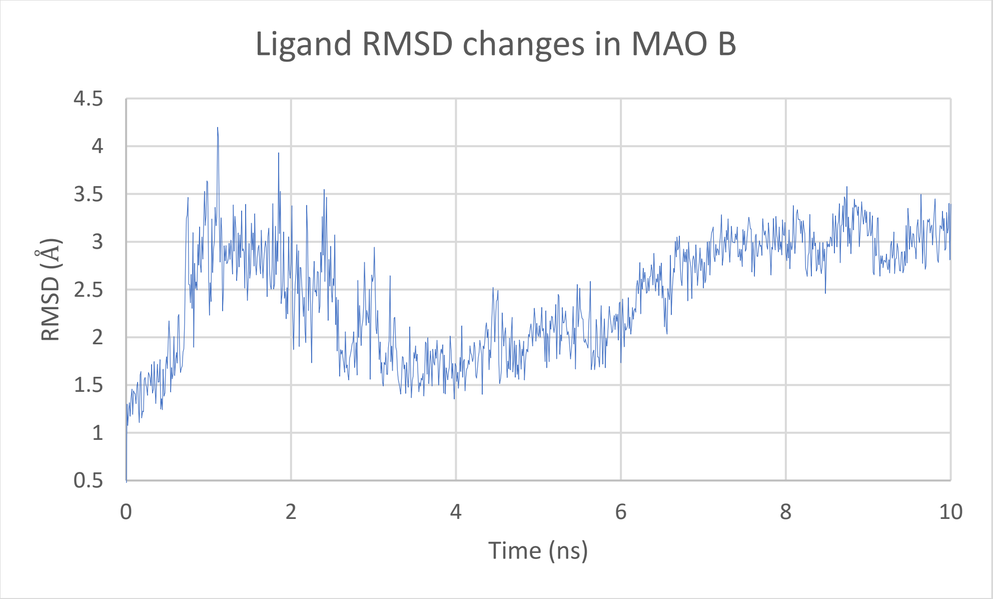

Figure S4. Changes of ligand **6** position within MAO B active site during molecular dynamics simulation

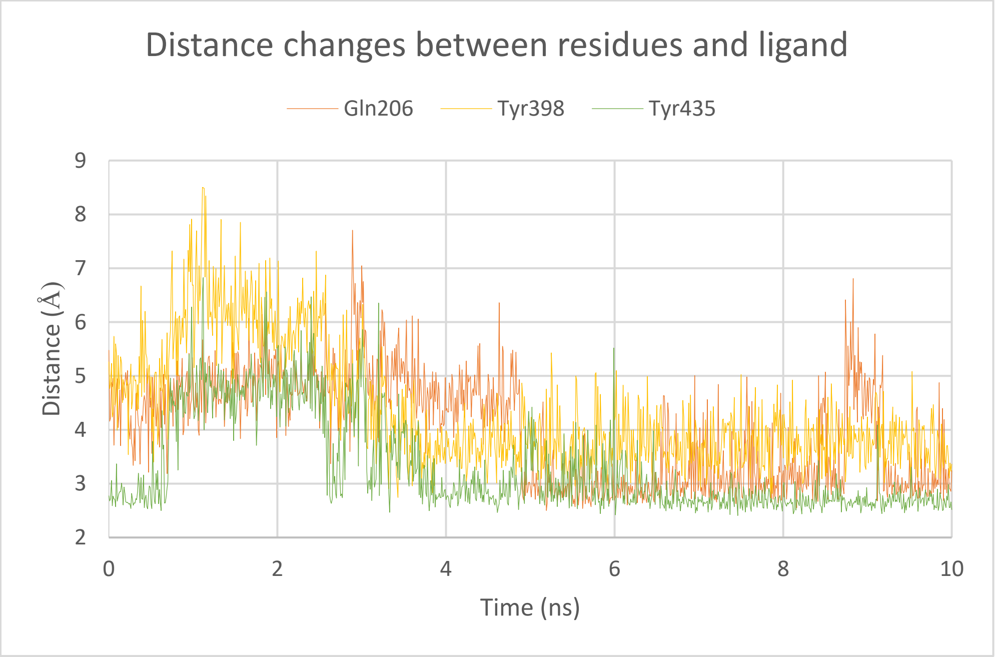

Figure S5. Distances between ligand **6** (CO group) and selected residues: Gln206(NH), Tyr398(OH), Tyr435(OH) from MAO B during molecular dynamics simulation

2. *In vitro* metabolic stability of compound 6

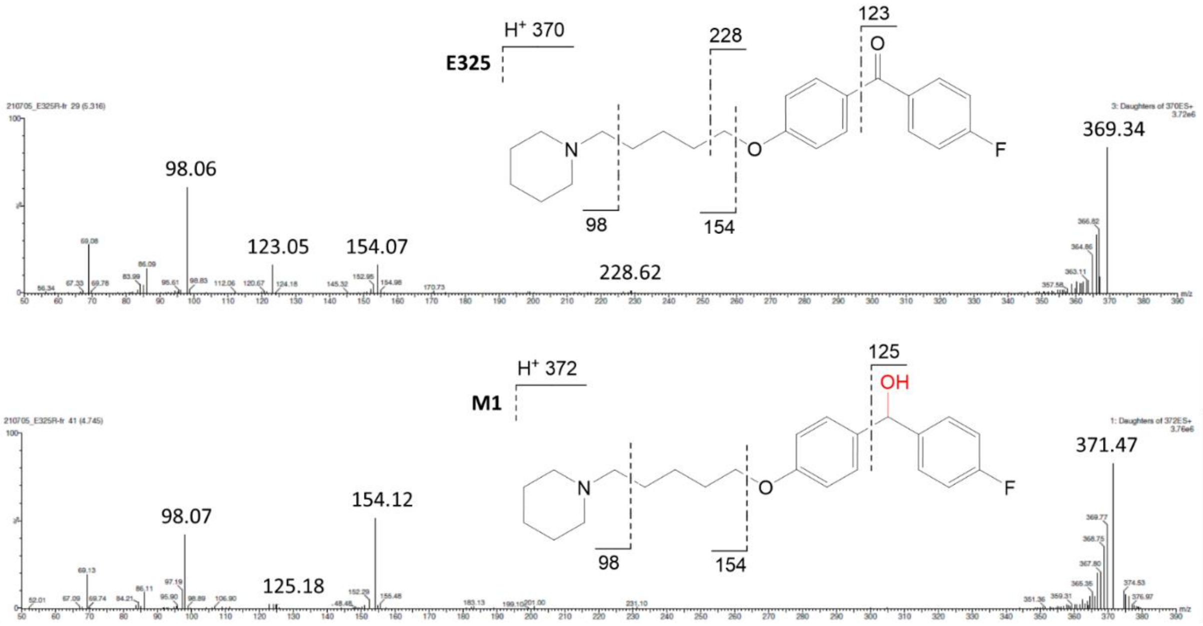

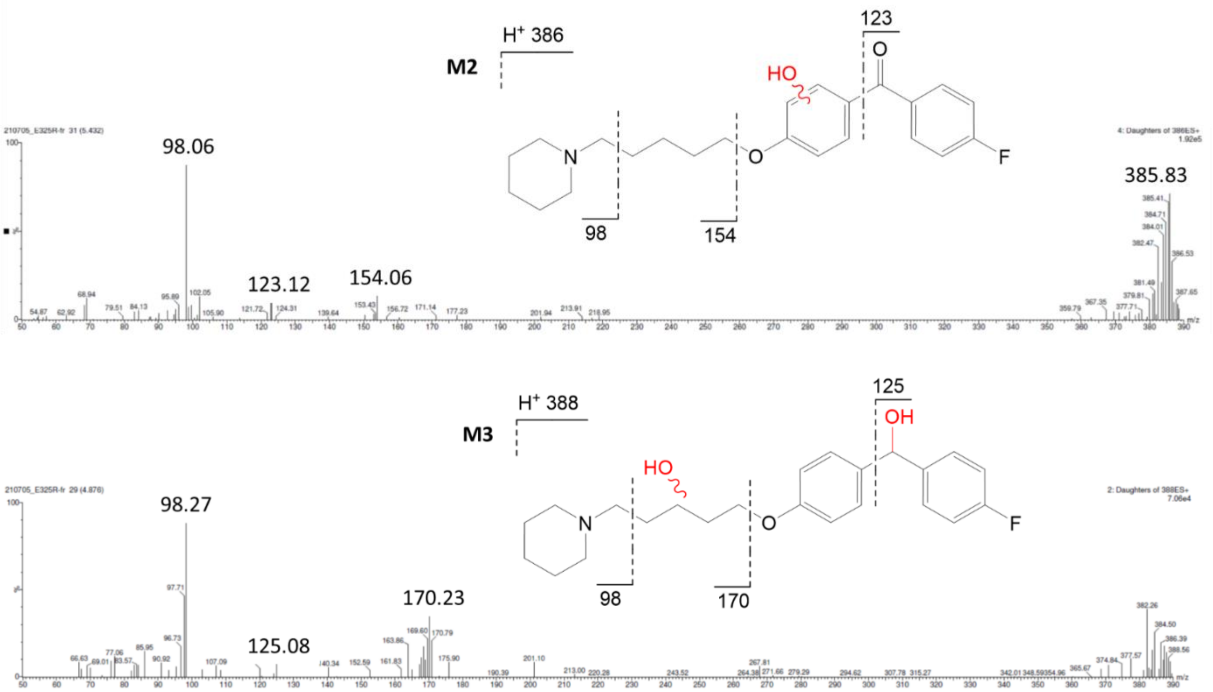

Figure S6. MS/MS ion fragmentation analyses of **6** and its metabolites.
